# One ancestor for two codes viewed from the perspective of two complementary modes of tRNA aminoacylation

**DOI:** 10.1186/1745-6150-4-4

**Published:** 2009-01-27

**Authors:** Andrei S Rodin, Eörs Szathmáry, Sergei N Rodin

**Affiliations:** 1 Human Genetics Center, School of Public Health, University of Texas, Houston, TX 77225, USA; 2 Collegium Budapest (Institute for Advanced Study), Szentháromság u. 2, H-1014 Budapest, Hungary; 3 Parmenides Center for the Study of Thinking, 14a Kardinal Faulhaber Str., D-80333 München, Germany; 4 Institute of Biology, Eötvös University, 1c Pázmány Péter sétány, H-1117 Budapest, Hungary; 5 Theoretical Biology, Department of Molecular Biology, Beckman Research Institute of the City of Hope, Duarte, CA 91010, USA

## Abstract

**Background:**

The genetic code is brought into action by 20 aminoacyl-tRNA synthetases. These enzymes are evenly divided into two classes (I and II) that recognize tRNAs from the minor and major groove sides of the acceptor stem, respectively. We have reported recently that: (1) ribozymic precursors of the synthetases seem to have used the same two sterically mirror modes of tRNA recognition, (2) having these two modes might have helped in preventing erroneous aminoacylation of ancestral tRNAs with complementary anticodons, yet (3) the risk of confusion for the presumably earliest pairs of complementarily encoded amino acids had little to do with anticodons. Accordingly, in this communication we focus on the acceptor stem.

**Results:**

Our main result is the emergence of a palindrome structure for the acceptor stem's common ancestor, reconstructed from the phylogenetic trees of Bacteria, Archaea and Eukarya. In parallel, for pairs of ancestral tRNAs with complementary anticodons, we present updated evidence of concerted complementarity of the second bases in the acceptor stems. These two results suggest that the first pairs of "complementary" amino acids that were engaged in primordial coding, such as Gly and Ala, could have avoided erroneous aminoacylation if and only if the acceptor stems of their adaptors were recognized from the same, major groove, side. The class II protein synthetases then inherited this "primary preference" from isofunctional ribozymes.

**Conclusion:**

Taken together, our results support the hypothesis that the genetic code *per se *(the one associated with the anticodons) and the operational code of aminoacylation (associated with the acceptor) diverged from a common ancestor that probably began developing before translation. The primordial advantage of linking some amino acids (most likely glycine and alanine) to the ancestral acceptor stem may have been selective retention in a protocell surrounded by a leaky membrane for use in nucleotide and coenzyme synthesis. Such acceptor stems (as cofactors) thus transferred amino acids as groups for biosynthesis. Later, with the advent of an anticodon loop, some amino acids (such as aspartic acid, histidine, arginine) assumed a catalytic role while bound to such extended adaptors, in line with the original coding coenzyme handle (CCH) hypothesis.

**Reviewers:**

This article was reviewed by Rob Knight, Juergen Brosius and Anthony Poole.

## Background

**Figure 1:**
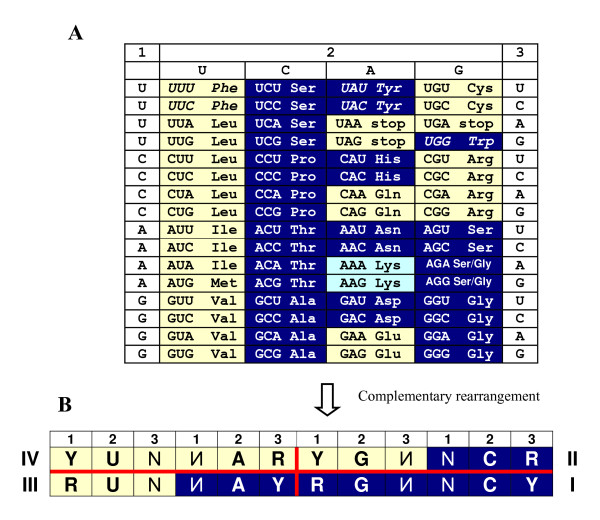
**The subcode for two modes of tRNA recognition by aaRSs**. (A) The conventional representation of the genetic code table with yellow and blue colors marking two modes of tRNA recognition by aaRSs – from the minor and major groove sides of the acceptor stem, respectively. Lys is colored in lighter shade of blue in order to indicate the fact that some archaebacteria use class I synthetases for this amino acid [9]. Stop codons are colored in yellow because the known cases of their "capture" by amino acids are mostly from class I [8]. Codons AGG and AGA are assigned to blue Ser or Gly, as they are in mitochondria (ibid.) Three aromatic amino acids, Phe, Tyr and Trp, with their mode of tRNA aminoacylation contradicting the class aaRS membership, are italicized. (B) The condensed rearranged table of the genetic code, in which complementary codons are put next to each other (all 32 pairs of complementary anticodons are shown in Figure 3). This rearrangement reveals the following rules of tRNA aminoacylation: (1) If the complementary codons contain YY *vs*. RR at the second and adjacent (either first or third) positions, their aaRSs recognize the tRNA acceptor from the same side of the groove, namely: minor (yellow) for 5'ИAR3' – 5'YUN3' pairs, or major (blue) for 5'RGИ3' – 5'NCY3' pairs; (2) If these positions are occupied by RY and YR, the modes of tRNA recognition are different, namely: minor (yellow) 5'YGИ3' *vs*. major (blue) 5'NCR3' and major (blue) 5'ИAY3' *vs*. minor (yellow) 5'RUN3'. These rules comprise the sub-code for two modes of tRNA aminoacylation that reveal four different quarters of complementary codons denoted by I, II, III and IV. Other symbols: N and complementary И denote all four nucleotides; R, purine (G or A); Y, pyrimidine (C or U). For details, see [11,12].

**Figure 2:**
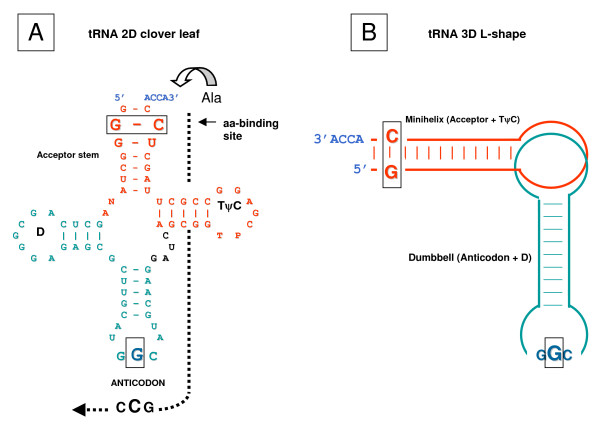
**Cloverleaf-like two-dimensional (A) and L-shaped three-dimensional (B) representations of tRNA structure**. The molecule shown is the *E. coli *tRNA^Ala ^with GGC anticodon and the 3G:U72 "wobbling" base pair that determine the identity of all Ala tRNAs across all species [2]. Because in the ancestral tRNAs with complementary anticodons the second bases in the acceptor are also complementary to each other [6,22], they are shown enlarged and boxed. The L-shaped tRNA consists of two halves, the minihelix (acceptor stem plus Tψ C arm), and the 'dumbbell' (anticodon arm plus D arm). The hypothetical aminoacylating ribozyme is shown by dotted line on the right (adapted from [12,24]).

The origin of the genetic code is a great challenge to evolutionists. The genetic code (Figure 1A) acts indirectly, through its adaptors (tRNAs). Each tRNA molecule has a CCA-3' end, to which the specific amino acid (aa) is attached, and an anticodon (the codon's complementary replica) that determines this specificity. However, these two sites are separated by nearly 70Å, the largest distance that is spatially possible within the tRNA molecule (Figure 2). These 70Å create many complications.

First, extant tRNAs cannot self-aminoacylate. Instead, 20 aminoacyl-tRNA synthetases (aaRSs), one for each amino acid, recognize and connect specific amino acids to the tRNAs, in accordance with the coding assignments (Figure 1A). However, the aaRSs are themselves proteins, proteins that mediate the translation of all protein-coding genes including... their own. This creates the proverbial "chicken-or-egg" problem. It is further exacerbated by the fact that mutations in aaRS genes, due to the special role played by the aaRSs in the translation process, accumulate at the accelerating rate. Apparently, to escape this error catastrophe [1], primordial life had no other means but to use the ribozymic precursors of the synthetases to aminoacylate tRNAs. We will refer to these hypothetical ribozymes as "r-aaRSs", to distinguish them from their isofunctional protein successors, "p-aaRSs".

Second, for at least ten amino acids, tRNA molecules truncated to the minihelix or the acceptor stem (or even a smaller fragment of the acceptor stem, with the CCA3' end) appear to contain enough "signal" for correct aminoacylation by cognate p-aaRSs ([2] reviewed in [3]). Likewise, truncation of the anticodon-binding domain in these p-aaRSs also did not change the specificity of tRNA charging (ibid). These experiments led to the hypothesis of the second (RNA "operational") code. The operational code is embodied in the acceptor stem and might have actually predated the "classic" code associated with anticodons ([4], see also [5,6]).

Third, p-aaRSs are divided into two classes (ten p-aaRSs each) that, although performing the same functions, have virtually nothing in common in their primary, 2D, and 3D structures [7]. However, p-aaRSs from different classes recognize tRNAs in a mirror, complementary, fashion: class I aaRSs approach the acceptor stem from the minor groove side and connect aas to the 2'OH hydroxyl of the terminal A76, whereas class II aaRSs approach the acceptor stem from the major groove side and connect aas to the 3'OH hydroxyl of the terminal A76.

The codon-to-aa assignment (Figure 1A) represents the universal genetic code, although deviations in some nuclear and organelle lineages being well known [8].

Amino acids usually also do not switch the class of their synthetases. The only known exception is LysRS – some archaebacteria use class I aaRS instead of the standard class II aaRS for this amino acid [9]. This suggests that potentially either of the two aaRS classes is versatile enough for specific aminoacylation in all 20 cases. Why, then, are there two classes? What advantage could that proffer? The recent analyses ([10-12], see also [13]) suggest the following (not mutually exclusive) answers: the distribution of the codon-to-aa assignments between the two modes of tRNA aminoacylation (1) facilitates the logically but not necessarily chemically attractive (see Results and Discussion section) gradual reduction of coding ambiguity and (2) protects complementary anticodons and cognate amino acids from translational confusion. In fact, a sub-code for the corresponding two modes of tRNA recognition exists – a sub-code that operates with complementary anticodons flanked by evolutionarily conserved 5'U and R3' nucleotides, and therefore has little if anything to do with p-aaRSs that often recognize solely the acceptor stem, and do not interact with the anticodon at all [11,12]. This suggests that the "yin-yang"-like pattern of two modes of tRNA aminoacylation and the corresponding sub-code (Figure 1B) are very ancient (definitely predating the origin of p-aaRSs of both complementary classes) and, as such, have been mediated by ribozymes. Obviously, such a ribozyme (r-aaRS) could easily recognize a single-stranded anticodon by Watson-Crick pairing. For 16 out of 32 pairs of complementary anticodons the risk of their confusion is minimal if r-aaRSs of two complementary types spread tRNA recognition in opposite directions away from their anticodons [11,12]. However, the (presumably) earliest amino acids, such as complementarily encoded Gly and Ala, were confusion-proof independently of whether their r-aaRSs recognized the anticodon loops in the same or the opposite directions [12]. This means that in the very beginning of development of the code, the choice between the two complementary modes of tRNA recognition did not depend much, if at all, on the anticodon loop.

In this report, we expand on the aforementioned analyses. In particular, we focus on the reconstructed ancestral acceptor stem, with the primordial operational code presumably located at the first three positions of the stem (Figure 2). This reconstruction reveals the palindromic symmetry of the acceptor stem. Accordingly, the very first complementarily encoded amino acids were "protected" from confusion when their ribozymic aaRSs both recognized the acceptor stem from the same (major groove) side. This finding strengthens the hypotheses that: (1) the two codes, operational and classic, had one ancestor [6,14,15] and (2) the code-shaping process predated even the origin of translation [16,17].

## Methods

8,246 tRNA gene sequences, representing the three domains of life, Bacteria, Archaea and Eukarya, were retrieved from the Genomic tRNA database [18].

We manually aligned sequences of concatenated tRNA genes and reconstructed the phylogenetic trees (see Additional files 1, 2, 3 and 4) using MEGA 4.0 phylogenetic analysis software . The tree topologies proved stable and sufficiently robust for our purposes (ancestral sequence reconstruction) with respect to the phylogenetic reconstruction method and substitution model used, and the Neighbor-Joining trees [19] obtained using Tamura-Nei distances [20] were selected as the baseline trees (please see the *Reviewers' comments *section for more detail on phylogenetic reconstruction). The trees were also consistent with the phylogenies obtained earlier [21], where five different whole genome-based approaches were used.

Subsequently, and separately for each anticodon, we manually reconstructed the ancestral tRNA sequences for each of these trees. Finally, we reconstructed the complete set of tRNAs for the hypothetical last universal common ancestor.

In the majority of the reconstructions (but not always – see Results and Discussions section below) ancestral second bases coincided with the consensus second bases [22] when we used the parsimony-based method [23] to reconstruct the ancestral states.

To measure the extent of the dual complementarity (DC), we counted the number of tRNA pairs with complementary anticodons in which the second bases of the acceptor helix were also complementary, and divided it by the total number of pairs [22]. Although the anticodons contain all four (G, C, A, and U) nucleotides, their presumed double-stranded homologs at the 1-2-3/70-71-72 positions of the acceptor stem almost exclusively use G and C (Table 1). Therefore, if the classic code embodied in the anticodons and the operational code embodied in the acceptor stem were independent of each other, then the expected DC value would be close to 0.5 (in reality, a little lower, since the rare U cases in the second acceptor position need to be accounted for). We used binomial distribution with p = 0.5 (two-tailed) to estimate statistical significance of the deviation of the observed DC values from the expected.

**Table 1:** Concerted complementarity of 2^nd ^bases in the acceptor stems of pairs of ancestral tRNAs with complementary anticodons^a^

Amino acids	Anticodons	2^nd ^bases in acceptor	Amino acids	Anticodons	2^nd ^bases in acceptor
	NAN × NUN			NGN × NCN	
1. Phe × Lys	**GAA × UUU**	**S × G**	17. Ser × Arg	**GGA × UCU**	**G × Y**
	**GAA × UUU**	**C × G**		**GGA × UCU**	**C × G**
	**GAA × UUU**	**C × C**		AGA × UCU	G/U × U/G
		C × G			S × S
		+			+
2. Phe × Glu	GAA × UUC	S × G/Y	18. Ser × Gly	GGA × UCC	G × C
	GAA × UUC	C × C		GGA × UCC	C × C
	GAA × UUC	C × C		GGA × UCC	G × C
		C × C			G × C
		-			+
*3. Leu × Stop * *(Leu × Gln)*	***UAA × UUG***	***C × G***	*19. Ser × Stop* Ser × Arg	***UGA × UCG***	***G × C***
	***UAA × UUG***	***C × G***		***UGA × UCG***	***C × G***
	***UAA × UUG***	***C × G***		***UGA × UCG***	***Y/G × G***
		C × G			S × S
		+			+
4. Leu × Gln	CAA × UUG	C × G	20. Ser × Arg	CGA × UCG	G × C
	CAA × UUG	C × G		CGA × UCG	C × G
	CAA × UUG	C × G		CGA × UCG	Y/G × G
		C × G			S × S
		+			+
5. Leu × Lys	AAG × CUU	C × G	21. Pro × Arg	**GGG × CCU**	**G × C**
	**GAG × CUU**	**C × G**		**GGG × CCU**	**G × G**
	AAG × CUU	G × C		AGG × CCU	G × C
		C × G			G × C
		+			+
6. Leu × Glu	GAG × CUC	C × G	22. Pro × Gly	GGG × CCC	G × C
	GAG × CUC	C × C		GGG × CCC	G × C
	GAG × CUC	G × C		GGG × CCC	x C
		S × S			G × C
		+ ?			+
*7. Leu × Stop*	**UAG × CUG**	**C × G**	23. Pro × Trp	UGG × CCA	G × G
*(Leu × Gln)*	***UAG × CUG***	***C × G***		UGG × CCA	G × G
	***UAG × CUG***	***G × G***		UGG × CCA	G × R
		C × G			G × G
		+			-
8. Leu × Gln	CAG × CUG	C × G	24. Pro × Arg	CGG × CCG	G × C
	CAG × CUG	C × G		CGG × CCG	G × G
	CAG × CUG	U × G		CGG × CCG	G × C
		C × G			G × C
		+			+
9. Ile × Asn	**GAU × GUU**	**G × C**	25. Thr × Ser	AGU × RCU	C × G
	**GAU × GUU**	**G × C**		**GGU × GCU**	**C × C**
	AAU × AUU	G × C/U		AGU × ACU	C × G
		G × C			C × G
		+			+
10. Ile × Asp	**GAU × GUC**	**G × C**	26. Thr × Gly	**GGU × GCC**	**C × C**
	**GAU × GUC**	**G × C**		**GGU × GCC**	**C × C**
	**GAU × GUC**	**G × C**		**GGU × GCC**	**x C**
		G × C			C × C
		+			-
11. Ile × Tyr^b^	**UAU × GUA**	**C × G**	27. Thr × Cys	**UGU × GCA**	**C × G**
	**UAU × GUA**	**x C**		**UGU × GCA**	**C × C**
	UAU × RUA	C × C		UGU × RCA	C × G
		ND			C × G
					+
12. Met × His	**CAU × GUG**	**G × Y**	28. Thr × Arg	CGU × ACG	C × G/C
	**CAU × GUG**	**G × C**		**CGU × GCG**	C × C
	**CAU × GUG**	**G × C**		CGU × ACG	C × G
		G × C			C × S
		+			- ?
13. Val × Asn	**GAC × GUU**	**G × C**	29. Ala × Ser	**GGC × GCU**	**G × G**
	**GAC × GUU**	**G × C**		**GGC × GCU**	**G × C**
	AAC × GUU	G × N		AGC × GCU	G × A/U
		G × C			G × N
		+			-
14. Val × Asp	GAC × GUC	G × C	30. Ala × Gly	GGC × GCC	G × C
	GAC × GUC	G × C		GGC × GCC	G × C
	GAC × GUC	x C		GGC × GCC	G × C
		G × C			G × C
		+			+
15. Val × Tyr	UAC × GUA	G - G	31. Ala × Cys	UGC × GCA	G × G
	UAC × GUA	G × C		UGC × GCA	G × C
	UAC × GUA	G × C		UGC × GCA	G × G
		G × S			G × G
		+ ?			-
16. Val × His	CAC × GUG	G - Y	32. Ala × Arg	CGC × GCG	G × Y
	CAC × GUG	G × C		CGC × GCG	G × C
	CAC × GUG	Y/G × C		CGC × GCG	G × C
		G × C			G × C
		+			+

DC value for the presumed common ancestor of Bacteria, Archaea and Eukarya:					
All pairs of complementary anticodons, i.e. NAN × NUN + NGN × NCN:					
Total: DC = 24/31 = 0.77, p = 0.003					
Stop codons excluded: DC = 21/28 = 0.75, p = 0.012					
Only strictly complementary pairing allowed: DC = 19/24 = 0.79, p = 0.006					

DC values for Bacteria, Archaea and Eukarya, all pooled together:					
					
NAN × NUN pairs:					
Total: DC = 34/44 = 0.77, p = 0.0004					
Stop codons excluded: DC = 29/38 = 0.76, p = 0.0016					
Only strictly complementary pairing allowed: DC = 17/24 = 0.71, p = 0.064					
					
NGN × NCN pairs:					
Total: DC = 31/46 = 0.67, p = 0.026					
Stop codons excluded: DC = 28/43 = 0.65, p = 0.066					
Only strictly complementary pairing allowed: DC = 23/31 = 0.74, p = 0.01					
					
All pairs of complementary anticodons, i.e. NAN × NUN + NGN × NCN:					
Total: DC = 65/90 = 0.72, p = 0.00002					
Stop codons excluded: DC = 57/81 = 0.7, p = 0.0004					
Only strictly complementary pairing allowed: DC = 40/55 = 0.73, p = 0.001					

^a ^Shown are the ancestral second bases for pairs of tRNAs with complementary anticodons in Bacteria, Archaea, Eukarya and their presumed common ancestors (ordered from top to bottom for each pair). Plus and minus signs denote presence or absence of the concerted second base complementarity, respectively. There is an apparent deficiency of anticodons beginning with 5'A, especially in the domains of Bacteria and Archaea. As complementary partners, one can use (in such cases) anticodons that begin with 5'G, which allows a wobbling G-U pairing (in bold). Similarly, one can also include the three pairs with "nonsense" anticodons (bold and italicized). The four cases of coevolutionarily maintained dual complementarity, where the second bases change in different kingdoms, yet remain complementary in both tRNA partners are underlined. Question mark behind + or - means that the corresponding assignment is likely, but not certain. "Incomplete" entry in columns 3 and 6 (such as "× C" in column 6 for pair 22, third line) means that the corresponding tRNA was not found in the database. See Methods section for a detailed explanation of how DC values were calculated.

^b ^This pair (Ile × Tyr) was not used in the DC calculations because its status is uncertain due to the small number of tRNAs available ("ND" standing for "non-determined").

DC values for the reconstructed common ancestor of Bacteria, Archaea and Eukarya are shown in the second panel of Table 1. A complete set of 32 codons was used to compute DC values, because the numbers for the separate NAN × NUN and NGN × NCN groups were too small to guarantee the robust estimation of statistical significance of observed differences.

It should be noted here that in statistical data analysis (in general) one is often faced with the dilemma of either pooling the subgroups together, or analyzing them separately. The primary advantage of the former is, of course, increased sensitivity, specificity and robustness of the analysis. However, if the subgroups are sufficiently heterogeneous, stratification (into subgroups) would be more appropriate. The obvious shortcoming of such stratification is smaller numbers within each subgroup, leading to the less robust statistical analyses. A less obvious (but equally insidious) problem is that sometimes it is unclear along which lines the data should be stratified (for example, should we perform a certain analysis separately for the three domains of life? Or pool Archaea and Eukarya together?) For the purposes of the above analysis, we decided that the benefits of pooling all codon type subgroups together far outweighed the disadvantages of stratification (namely, numbers so low as to severely compromise the robustness of the binomial testing).

Similar reasoning applies to DC values for Bacteria, Archaea and Eukarya (shown in the third panel of Table 1). In this analysis, the three domains were pooled together because not only there was no *a priori *evidence suggesting subsample heterogeneity (with respect to DC values), but an argument can actually be made that there is even *less *stratification, as far as dual complementarity is concerned, than one would generally expect for the three domains. Indeed, direct cases of maintained dual complementarity, in spite of high variability of the second bases in various Bacteria, Archaea and Eukarya lineages (down to the different species level), suggest that the dual complementarity maintenance phenomenon is largely invariant across (hence, independent of) the domains of life. Therefore, stratifying by domains does not make much sense, and might even prove harmful through its arbitrariness.

## Results and discussion

### Complementarity-based evolutionary link between the acceptor stem and anticodon

The independence of the operational and classic codes leads to certain irreconcilable contradictions (detailed in [6,22]). Ever since the experiments uncovering the operational code were performed [2], it was obvious that the simplest solution of the entire problem would be to have an anticodon/codon homolog in the acceptor stem, as close as possible to the CCA-3' end [5,14,15]. The optimal location would be the very first 1-2-3/72-71-70 positions of the acceptor stem [6,15], which largely determine the identity of tRNAs. To the best of our knowledge, all attempts to find any such straightforward homology have failed. And yet, it is possible to observe still extant vestiges of the ancient duplication when one considers the pairs of consensus tRNAs with complementary anticodons: these tRNAs turned out to be complementary at the second position of the acceptor as well ([6], updated in [22]). The ancestral tRNAs reconstructed from the phylogenetic trees of Bacteria, Archaea and Eukarya [24] strongly confirmed this finding (depicted in Table 1 for all pairs of ancestral tRNAs with complementary anticodons). It should be noted that the DC values were miscalculated in [25]. The primary reasons for the discrepancy were mis-determination (pairs 4, 18 in Table 1), mis-interpretation (pair 32) and omission (pairs 9, 17, and 21). Additionally, the ancestral bases reconstructed from the phylogenetic trees (such as the ones used in this analysis) are more accurate than the simple consensus majorities, especially if the number of sequences is small (only 1,100 sequences were used in [25]).

Worth noting are the pairs (underlined in Table 1) in which one "partner" has different second bases in the acceptor stem in different domains (for example, "G" in Bacteria and "C" in Archaea) and the other partner possesses the domain-specific complementary second bases ("C" in Bacteria and "G" in Archaea, respectively). This striking dual complementarity, which is maintained despite the variability in the second bases, is observed not only with the different domains but also even among different species within a domain. The pairs Val(GAC) × Asp(GUC) in bacteria and Ser(AGA) × Arg(TCT) in eukaryotes are good examples (Table 1: pairs 14 and 17). Furthermore, we show below that the dual complementarity is causatively associated with the sub-code for the two modes of tRNA aminoacylation. In fact, it is the sub-code that explains away rare deviations from dual complementarity.

Dual complementarity was detected for pairs of ancestral tRNAs with completely complementary anticodons and was not detected for pairs of tRNAs in which only the central bases of anticodons were complementary [22]. This important difference suggests that the dual complementarity not only indirectly reflects the common ancestry of operational and classic codes, but also might imply that the ancestral code recruited new codons by complementary pairs (as opposed to one-by-one). There are at least two possible mechanisms for such recruitment: (1) through primitive in-frame translation from both complementary strands (before their differentiation into the sense and antisense strands) [6,11,12,22,26,27] and/or (2) utilization of the complementary pre-tRNA strands arising through replication anyway [6,28,29], possibly in the role of novel coenzyme (aa)-binding handles [16,17]. A pair of complementary 'codons' that was already captured by the evolving code would then generate (through complementary mutations) the next, "daughter" pair. Accordingly, we suggested two tetrads of amino acids that, by fitting the above mechanism in the most parsimonious fashion, might be the best candidates for the very first amino acids recruited by translation [22]. These are [Ala(GCC), Gly(GGC), Val(GTC), Asp(GAC)] and [Ala(GCG), Arg(CGC), Val(GTG), His(CAC)] (codons are shown in parentheses). As we shall see below, this set has remarkable potential for building simple proteins.

The complementary pair-based scenario of early code shaping renewed the interest in the division of p-aaRSs into two classes, and suggested they originated from the complementary strands of the same ancestral gene [30-32]. The two classes of p-aaRSs coincide almost perfectly with the two modes of tRNA aminoacylation, from the major and minor groove sides of the acceptor helix (shown in Figure 1 in blue and yellow, respectively). Out of 20 amino acids, only three (all aromatic), Phe (class II), Tyr (class I) and Trp (class I), have a mode of tRNA aminoacylation that is associated with the opposite class. Another important piece of the puzzle is that p-aaRSs of both classes are evolutionary latecomers among paralogous proteins [33], which strengthens numerous arguments in support of the hypothesis that, before the p-aaRSs advent, ribozymes acted as synthetases [16,17,34,35], including both direct experimental evidence [36] and the line of reasoning stemming from the two mirror symmetric modes of tRNA recognition by p-aaRSs [11,12]. Finally, it is remarkable that certain other protein families also reveal this complementarity. Class I p-aaRSs have obvious homology to NAD^+^- and NADP^+ ^– dependent dehydrogenases of the Rossmann fold [7], class II p-aaRSs are homologous to the HSP70 family [31], and it appears that in *Achylia klebsiana *a single gene encodes for an HSP70-like chaperonin and a glutamate dehydrogenase *via *its sense and antisense strands, respectively [31].

### Sub-code for two aminoacylations

The major *vs*. minor groove side contemplation of the genetic code revealed the symmetry (Figure 1A) that prompted us to re-arrange the code table by putting complementary codons next to each other. This re-arrangement uncovered the "yin-and-yang" pattern (Figure 1B) that represents the otherwise latent sub-code for the two modes of tRNA aminoacylation.

**Figure 3:**
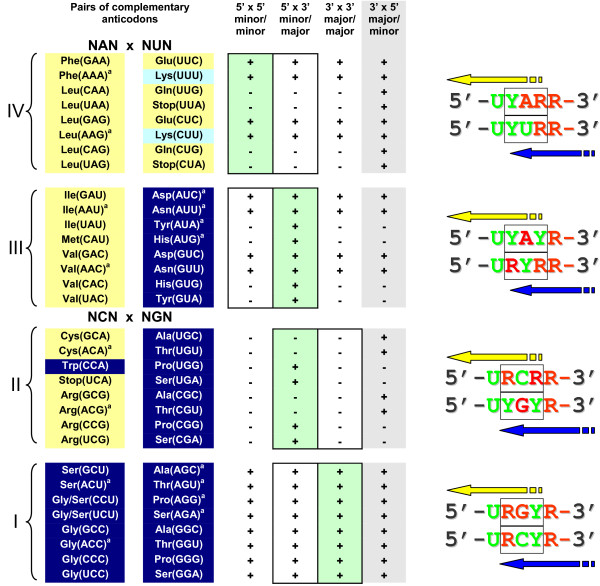
**Risk of confusion of complementary anticodons (at the purine/pyrimidine R/Y resolution) under four scenarios of tRNA recognition by two putative r-aaRSs (adopted from **[12]**).** Pairs of complementary anticodons are ordered following the "yin-yang" pattern of Figure 1B. Plus signs denote the pairs that have no identical tetra(or more)- nucleotides within the loop 3' YU-XYZ-RN5' --- that is, they are distinguishable (under the corresponding scenario) by two putative ribozymes that recognize the complementary tRNA halves. Minus signs mark the contrary, indistinguishable, cases. For each pair, only a zero- or one base-long shift in one of two directions from the anticodon is allowed. Two simultaneous shifts (one in each anticodon loop) are considered highly unlikely. All 32 pairs are divided in four quarters (enumerated I, II, III, and IV) according to the sub-code for two complementary modes of tRNA aminoacylation. The earliest quarter is supposed to be I, the latest, IV (see text for detail). The quarters II and III include 16 RY vs. YR-type pairs that satisfy the second rule of the subcode for two aminoacylations (see legend to Figure 1B). If, following Figure 4, we assign NGN and NAN anticodons to major and minor groove sides, this would exclude the entire 3' × 5' scenario (shaded) and the number of conceivable scenarios of tRNA recognition is reduced from four to only two (enclosed by rectangles). The actual evolutionary pathway is shown in green. ^a^5'ANN3' anticodons usually do not exist – instead, the 5'GNN3' anticodons recognize not only the legitimate 3'CИИ5' codons but also the illegitimate wobbling 3'UИИ5' codons. For the strictly legitimate nine pairs of the RY vs. YR type, the +/- ratio is 7:2 (second scenario) vs. 3:6 (fourth scenario) – the former being, therefore, seven times more "secure". Pairs of pentanucleotides 5'U-XYZ-R3' on the right (with complementary anticodons in the center) show the risk of confusion by r-aaRSs under the 5' × 3' scenario of recognition for the corresponding pairs from quarters I, II, III and IV. Compare the risk of confusion for I vs. IV and II vs. III (see text for detail).

**Figure 4:**
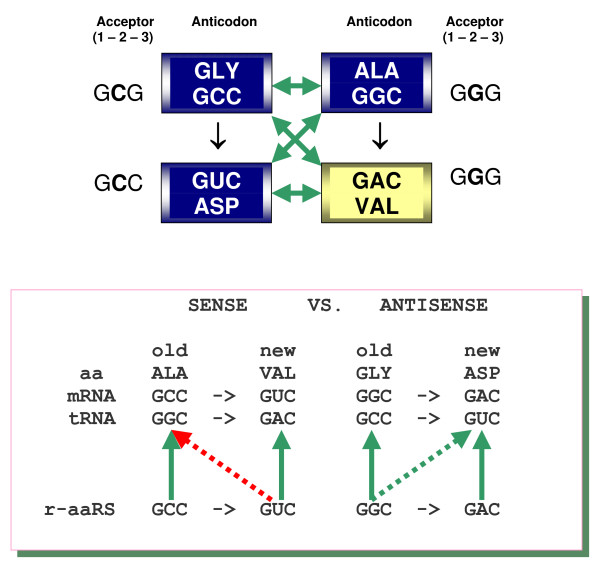
**The possible first tetrad recruited by the genetic code (top panel) (reviewed in **[26,27]). Consistent with our model, these four amino acids show dual complementarity not only in the legitimate Gly(GCC) – Ala(GGC) and Asp(GUC) – Val(GAC) pairings but also in the Gly(GCC) – Val(GAC) and Ala(GGC) – Asp(GUC) pairings, with G-U and even A*C mismatch at the central position [22]. Accordingly, the Ala-Gly pair could generate the new Val-Asp pair via C→U and G→A transitions (ibid.) The Ala→Val expansion of the code is accompanied by a change of tRNA recognition from the major (blue) to the minor (yellow) groove side, whereas the complementary Gly→Asp leaves the mode (blue) unchanged. This asymmetry could be associated with a risk of pleiotropic tRNA mis-aminoacylations by ribozymes. The schematic below (under the tetrad) (adapted from [12]) demonstrates this risk for Ala→Val and Gly→Asp expansions with legitimate (solid arrows) and 'wobbling' (dotted arrows) recognitions of anticodons by r-aaRSs. The Ala(GCC) → Val(GUC) expansion is prone to multiple mis-aminoacylations of the old tRNA^Ala ^by the new r-ValRS (red dotted arrow). In contrast, the complementary Gly(GGC) → Asp(GAC) expansion has no such disadvantage (green dotted arrow). To escape potential pleiotropic complications a different mode of tRNA recognition is required that would safely distinguish the r-ValRS from the already established r-AlaRS. This is precisely what happened: AspRS preserved the (same as GlyRS) recognition from the major groove (blue), whereas ValRS adopted the recognition from the minor groove (yellow).

Among many possible codes, the real genetic code (Figure 1A) shows a relatively high immunity to the effects of mutations and reading errors [37]. The sub-code of aminoacylation (Figure 1B) adds to that by minimizing the risk of confusion for 16 pairs of complementary anticodons that contain YR palindromes [12,13]. The "yin-and-yang" pattern is perfectly symmetrical as far as switching the "colors", i.e. the modes of tRNA aminoacylation (major groove "blue"/minor groove "yellow", see above), is concerned (Figure 1B). Therefore, the risk of confusion might seem to be invariant with respect to this color/mode switch. However, if one takes into account not only the anticodons *per se*, but also the adjacent U and R (from the 5' and 3' sides, respectively), the asymmetry of the risk of confusion is immediately revealed (Figure 3). Here we highlight the following aspects of the asymmetry:

1. The eight (blue/blue) pairs of complementary anticodons in quarter I of Figure 3 are associated with abiotically synthesizable ("Miller") amino acids [38], presumably evolutionarily early ones, including the likely earliest Ala (GGC) × Gly (GCC) pair [26,27]. Remarkably, none of the eight can be confused under any of the four scenarios (Figure 3): both yellow (5' × 5'), both blue (3' × 3'), yellow × blue (5' × 3'), and blue × yellow (3' × 5'), *as if the anticodon did not play any role at all*. The possible reason for this anticodon-indifference is shown on the right (Figure 3). The configuration of pyrimidines (Y) and purines (R) for these pairs, i.e. 5'U-RRY-R3' × 5'U-RYY-R3', does not allow confusion in aminoacylation under any of the four scenarios. The robustness of these 5'U-RRY-R3' × 5'U-RYY-R3' blue pairs is especially remarkable when contrasted with the analogous 5' U-YRR-R 3' × 5' U-YYR-R 3' yellow pairs in quarter IV of Figure 3. The latter are apparently sensitive to misreading by the primordial synthetases. Therefore, it might not be a coincidence that two of the four confusion-prone triplets, UUA and CUA, are used as the translation stop signals [12].

2. In addition to the quarter I, there are eight other pairs of complementary anticodons in quarters III and IV that cannot be confused under any of tRNA recognition scenarios (Figure 3). Interestingly, the pairs Asp(GUC) × Val(GAC) and Glu(CUC) × Leu(GAG), often suggested to be among the earliest amino acids [26,27,38] fall into this group.

3. Recognition of anticodons by r-aaRS ribozymes was likely based on the complementary base pairing [16]. With regard to G-C *vs*. C-G or A-U *vs*. U-A, the recognition is symmetric. Why then was the recognition *via *the major groove side assigned to the second column of the code table (Figure 1A), i.e. central C in codons (G in anticodons), and not to the complementarily symmetric fourth column? The same question arises for the first and third columns. A possible explanation: the first and second columns might have been selected for the minor and major groove sides of tRNA recognition because the risk of confusion of complementary anticodons was much lower than under the seemingly symmetric choice of third and fourth columns (Figure 4). In fact, this difference is rooted in the fundamental asymmetry between the wobbling G-U (still a pair) and complementary A*C (a clear mismatch) (Figure 4; see ref. [12] for details).

4. The differentiation of NYN codons into the major groove (blue) NCN and minor groove (yellow) NUN codons narrows the repertoire of subsequent evolutionary routes to the pathway that has been actually chosen (shaded green in Figure 3) by leaving only two options for each type of complementary pairs (framed in Figure 3), out of the conceivable four. In fact, this differentiation makes the fourth scenario, 3' × 5', very unlikely, and the advantage of the second scenario, 5' × 3', even more substantial (see [12] for details). Remarkably, this advantage is associated with amino acids of high catalytic propensity such as His, Arg, etc. [39]. Furthermore, these are the very amino acids for which the cognate triplets (anticodons and/or codons) occur more frequently in the aa-binding centers of RNA aptamers than expected by chance alone, thus suggesting that stereochemical affinity played a part in shaping the code [40-42]. We think that it is not a coincidence that the risk of confusion for such "Yarus" amino acids from the minor groove/major groove (yellow/blue) 5' × 3' pairs depends so much on the anticodons with adjacent 5'U- and -R3' (in contrast to "Miller" amino acids from the major groove/major groove (blue/blue) 3' × 3' pairs).

The anticodon-flanking 5'U- and R-3' nucleotides appear to determine the integral pattern of confusion risk for the pairs of complementary anticodons shown in Figure 3. If, for example, one flips these U and R nucleotides, the situation is reversed: the complementary anticodon loops 5'R-YYR-U3' and 5'R-YRR-U3' become the confusion-resistant pairs, in contrast to the 5'R-RRY-U3' and 5'R-RYY-U3' pairs, which now become confusion-prone (under either of the tRNA recognition modes). However, in nature these 5'U and R3' are conserved throughout all tRNAs. Therefore, we speculate that during the genetic code shaping process, natural selection favored the pairs of complementary anticodons "adapted" to the already existing adjacent nucleotides.

The above speculation makes even more sense if one takes into account that it is precisely the 5'U- and -R3' flanks of RRY (and complementary RYY) anticodons that could have provided smooth, sequential reading of codons in the primitive translation ([43] see also [26]). In the evolutionary beginning of the translation the amino acid assignments might not have been as specific as they became later – what was important was the translation process in itself, and the availability of amino acids. Regardless, this configuration of anticodon loops possesses yet another substantial advantage: the lowest possible (zero, actually) risk of confusion of complementary anticodons for the presumed early amino acids (Figure 3). It is important to realize that this advantage might have been crucial in the advanced RNA world, where ribozymes used amino acids as cofactors [16,17]. Note in this regard that, as a rule, pairs of complementary triplets encode the functionally very different amino acids, most often those with a high catalytic propensity (His, Asp, Glu, Lys, Arg) contrasted with those with a low catalytic but high structural (beta sheet building) propensity (Val, Ile, Leu, Phe, Ala) [39]. Therefore, it is clear that confusion of these complementarily encoded amino acids would be of a great disadvantage for riboorganisms. And, accordingly, *it would be of a great advantage for the RNA-based life to utilize the 5'U-XYZ-R3' configuration of the anticodon loop and the sub-code for two modes of tRNA aminoacylation even before the origin of translation *per se. This idea will be comprehensively discussed elsewhere [44]. Here we address the following questions:

(1) Where from (and when) did these important conserved 5'U- and -R3' flanks of anticodons come?

(2) For the 3' × 3' pairs of the first ("Miller") amino acids, their anticodons contribute little (if anything) to the sub-code for the two modes of tRNA recognition that protects the tRNA pairs from mis-aminoacylation. Might this imply that the recognition of these amino acids is encoded elsewhere in the tRNA molecules?

(3) Why was the 3' × 3' option chosen if the three other options (5' × 5', 5' × 3' and 3' × 5') seem to be equally error-proof? Was this choice random or was(were) there any selective advantage(s) behind it?

### Primordial palindrome of tRNA

**Figure 5:**
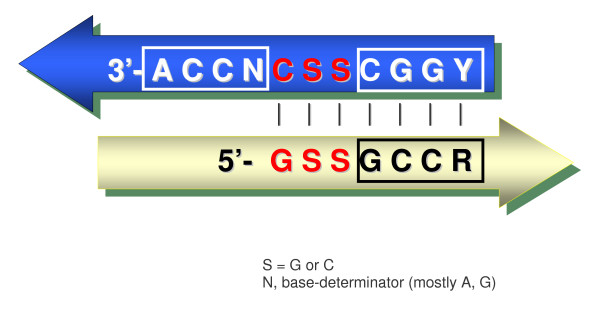
**Acceptor arm for the tRNA last universal common ancestor (LUCA)**. Shown in red is the triplet that, according to [15] and the dual complementarity hypothesis [6], was originally the duplicate of the anticodon. Yellow and blue mark the two modes of tRNA recognition by aaRSs, from the minor groove (5') and major groove (3') sides, respectively. The terminal NCCA, its presumable homolog GCCR and complementary YGGC quadruplets are framed. Note that the blue (3') half of the structure is a palindrome.

Searching for the answers to these questions inevitably brings us to the tRNA acceptor stem. Although the acceptor stem is highly variable, especially at the 4-5-6, positions [45], we reconstructed its common ancestor (Figure 5) from the phylogenetic trees for Bacteria, Archaea and Eukarya (see Materials and Methods section above). The 5' part of the acceptor is a heptamer that consists of a proto-anticodon (or codon) triplet (according to the dual complementarity) and a quadruplet GCCR that is homologous to the universal NCCA 3' tail. Then, the 3' counterpart appears as a palindrome with a codon-like triplet in the middle (Figure 5).

**Figure 6:**
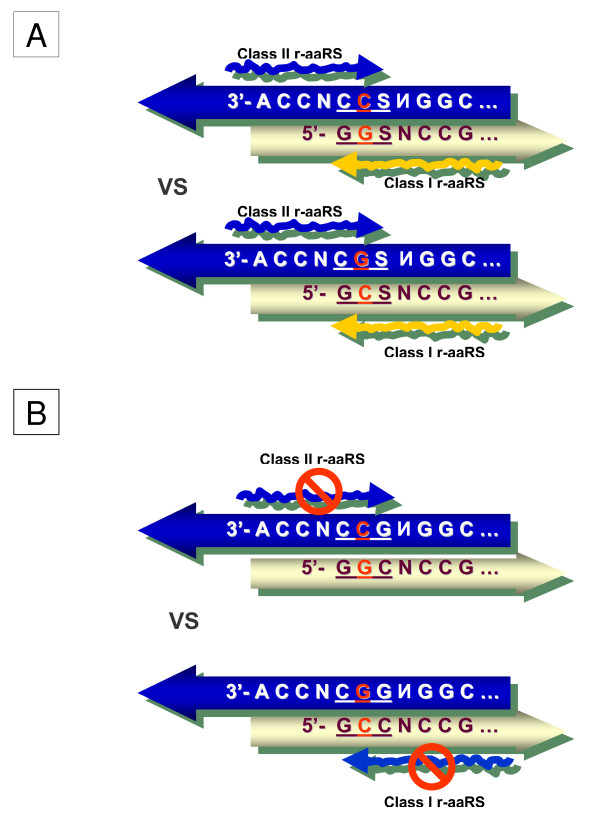
**(A): Ancestral acceptor stems of tRNAs that have complementary second base pairs (shown in red), in accordance with dual complementarity (Table 1)**. The putative r-aaRSs are shown as wavy arrows. "S" stands for either G or C. These two acceptor stems could have existed during the earliest stages of code shaping, even predating translation (i.e. before the pairs of single-stranded anticodons at the bottom of Figure 3 came into existence). (B): The same acceptor stems as in (A), but with the mutually complementary GGC/GCC and GCC/GGC pairs in the center. "Prohibited" denotes more confusion-prone recognition of acceptors by r-aaRSs.

Two such acceptor stems with complementary second bases (G and C shown in red) within the triplets that might have had a common ancestor with anticodons are shown in Figure 6. It should be noted that in modern tRNAs the presumable codon/anticodon-like motifs that are most frequent at this location correspond to Gly, Ala, and Pro (all "major groove" amino acids), with the next most common being Arg (a "minor groove" amino acid) (see Figure 4 in [22]).

In our model, the first position of the acceptor corresponds to the first position of the anticodon that, in turn, corresponds to the degenerate third position of codons. Not surprisingly, because it is not essential for encoding amino acids, this position in the acceptor is almost invariably occupied by the G-C base pair, "possibly for structural reasons to stabilize the end of the acceptor helix" [46]. The second position of the acceptor is supposed to be the most important position – both in the primordial operational code, and in the genetic code (represented by anticodons in tRNAs and codons in mRNAs) – for encoding group properties of similar amino acids. The third position of the acceptor stem corresponds to the third position of the anticodon that, in turn, corresponds to the first position of codons, which is important for further, more specific, coding. The classic example is the Ala-specific G-U base pair at this position (Figure 2) [2,4].

The symmetric location of the anticodon precursor at the 70-71-72 positions of the opposite 3' strand of the acceptor stem also makes sense [15]. At any rate, it seems reasonable to assume that the base pairs in the second position of the acceptor stems made the most difference in primordial RNA-mediated aminoacylation.

Importantly, to avoid confusion between two acceptor stems with complementary second bases, both putative r-aaRSs must use the same groove side for tRNA recognition, i.e. major groove/major groove (3' × 3') or minor groove/minor groove (5' × 5') scenarios (Figure 6A). In these cases, the r-aaRSs encounter different second bases, thus avoiding wrongly charging the acceptors with "complementary" amino acids. This is not the case under the (3' × 5') or the mirror (5' × 3') scenarios.

Furthermore, let us consider the two acceptor stems in which the GSS/SSC configuration at the 1-2-3/70-71-72 positions (Figure 5) is represented by the complementary palindromes GCC/GGC and GGC/GCC, respectively (Figure 6B). A closer inspection of these two acceptor stems reveals that the errorless (5' × 5') scenario of their recognition does not differ from the (3' × 3') scenario. Indeed, the two putative r-aaRSs (which recognize the acceptor stem from the major and minor groove sides) are facing identical sequences (Figure 6B). Accordingly, these two r-aaRSs were probably very much alike themselves. Thus, for complementary GCC and GGC triplets, the palindrome symmetry of the ancestral acceptor stem makes the primordial two classes of r-aaRSs, and their modes of recognition, functionally undistinguishable. The difference between these modes of recognition does materialize, but only if the proto-tRNAs gain, and incorporate into the recognition process, the single-stranded anticodon loop of the 5'U-XYZ-R3' configuration. All this might explain why out of four options (Figure 3) it was the 3' × 3' scenario that was "chosen" – in reality, there was simply nothing to choose from!

At the same time, because the 3' strand of one acceptor stem and the 5' strand of the other acceptor stem in Figure 6B are practically undistinguishable, each of the two acceptors faces an increased risk of being recognized from the opposite side by the complementary r-aaRS, thus inviting erroneous aminoacylation. The risk appears all the more real in light of the following considerations:

1. Substantial evidence points to the complementarily encoded Gly (GCC) and Ala (GGC) as the very first pair of amino acids that were recruited by the genetic code;

2. These two triplets are often found at the 1-2-3 positions of the acceptor stem in tRNAs, and yet...

*None of the pairs of ancestral tRNAs with complementary anticodons (including tRNA^Gly ^and tRNA^Ala ^with GCC and GGC, respectively) has GCC and GGC at the 1-2-3 site of the acceptor*. This inconsistency can be explained by an increased probability of erroneous aminoacylation for the GGC/GCC pair (Figure 6B). In fact, the complete absence of GGC/GCC triplets in the acceptor stems of tRNAs with complementary anticodons is another strong argument in support of the decisive role of the acceptor stem in the early stages of the genetic code evolution.

The (3' × 3') scenario efficiently discriminates the acceptors with complementary second bases. The symmetric (5' × 5') scenario also appears to discriminate; however, the presence of an unpaired NCCA-3' makes the (3' × 3') scenario more preferable for recognition by r-aaRSs.

This primary, anticodon-independent, choice of the (3' × 3') scenario suggests that:

1. The operational code embodied in the acceptor stem is very ancient, and the second base pair was originally the only (and therefore the most important) base pair in coding.

2. Initially, the operational code was activated through the degenerate, one-mode (from the major groove side) recognition of the acceptor – recognition that predated the sub-code for two modes of aminoacylation. The sub-code was established later when, as a result of duplication, the single-stranded anticodon loop emerged.

3. The tRNA molecule might have started its evolution into the extant cloverleaf structure from the acceptor arm [4,47-49], although the nearly perfect symmetry of the cloverleaf with the anticodon in its center makes the anticodon-first scenario also feasible [17].

We will compare the acceptor-first and anticodon-first models elsewhere [44]. Here we would like to emphasize that in any case the primary (3' × 3') "choice" in the acceptor stem turned out to be very "fateful" – it effectively ruled out the (3' × 5') scenario (Figure 3: shaded column), thus predetermining the subsequent genetic code shaping (see [12] for detail).

According to the acceptor-first model of tRNA origin [4,6], the invariant 5'U- and -R3' flanks of anticodons came from the initial palindrome as the homologs of the base-determinator N and its complement (И). Fittingly, the most frequent base-determinator is A (the second most frequent being G), and its complement is U (note that U can form complementary pairs with G as well). Remarkably, it is precisely these invariant U and A (sometimes G) bases that adjoin the anticodon from the 5' and 3' sides, respectively. Therefore, it might not be a coincidence that when (in some genomes) the tRNAs are encoded in pieces, the major site of introns, splits on minigenes [50,51], and processing in permuted tRNA genes [52] is located between the 37^th ^and 38^th ^nucleotides. This is the position that makes the lowest-confusion-risk (5' × 3') scenario of tRNA aminoacylation by two putative r-aaRSs work, especially if these r-aaRSs are located in introns, in close proximity to the 37^th ^nucleotide [16,17,53]. Furthermore, consecutive duplications of the original palindrome (by self-priming and self-templating) could produce the tRNA cloverleaf of a full length (see Additional file 5). This cloverleaf has many of the extant tRNA’s invariant and largely invariant nucleotides as well as sites of splitting tRNA in pieces (see [50-52]). The sites are often located next to N and И, following the former or preceding the latter.

### The sub-code of aminoacylation is correlated with dual complementarity

The nature of selection is that it is strictly a tactical force – it knows of no long-term strategy and has no foresight to meet the future demands [54]. From this viewpoint, there is a crucial difference between the operational code mediated by p-aaRSs and its remote ancestor mediated by r-aaRSs. Obviously, the enzyme p-aaRSs, even in their primal simplicity, could not have appeared before translation. On another hand, since nothing in nature evolves with foresight, the putative ribozyme r-aaRSs not only could but must have emerged before translation and, as shown in Figs. 5 and 6, this primordial ribozyme-mediated operational code in the acceptor stem could indeed predate the translation associated with anticodons. Therefore, it appears that the leading role in maintaining the specificity of tRNA aminoacylation must, at some point, have passed from the acceptor stem to the anticodon and then, after the genetic code established its complementary core, must have returned back to the acceptor stem (accompanied by the r-aaRS → p-aaRS transition). This sequence of events is reflected (approximately bottom-to-top) in Figure 3. Worthy of emphasis here is the evolutionary continuity that preserved (well) the two complementary modes of tRNA recognition by aaRSs while undergoing the ribozyme → enzyme reinvention.

The sub-code for the two modes of tRNA aminoacylation appears to have little to do with complementary anticodons in two situations: (1) when the anticodon loop and stem had not yet emerged (Figs. 5, 6), and (2) when the anticodons existed, but they had a high risk of confusion under most of the scenarios of tRNA recognition (meaning "three minuses" in Figure 3, columns 3–5). If, in the first situation, the same mode of recognition of ancestral acceptor stems (major groove/major groove) with complementary second base pairs helped to decrease mis-aminoacylation as Figure 6 suggests, then one would predict for the second situation that, symmetrically, the acceptor stems with the same second base pair need different modes of recognition to minimize the risk of confusion. However, having the same second base pair means a lack of dual complementarity. Interestingly, in major groove/minor groove YGN × ИCR pairs of anticodons with a "three-minus" risk of confusion (quarter II in Figure 3) dual complementarity is absent (Ala(UGC) × Cys(GCA) and Thr(CGU) × Arg(ACG)) or indistinct (Thr(UGU) × Cys(RCA)). This can be compared to the perfect dual complementarity of Leu(CAR) × Gln(YUG) pairs (quarter IV in Figure 3) – these pairs are also "three-minus", but in contrast to the aforementioned major groove/minor groove YGN × ИCR pairs, Leu(CAR) and Gln(YUG) are both recognized from the same groove (minor). Having complementary second bases in their acceptor stems would protect them from being incorrectly recognized by aaRSs, and this is exactly what we observe.

Also relevant is that the first and third columns of the genetic code table (NAN and NUN anticodons) reveal the dual complementarity much better than the second and forth columns (NGN and NCN anticodons) (Table 1, see also [22]). This difference can be explained if one takes into consideration that the sub-code for the two modes of tRNA recognition discriminates the minor groove/major groove NAY × RUN pairs (quarter III in Figure 3) better than their major groove/minor groove YGN × ИCR counterparts (quarter II in Figure 3).

Dual complementarity of the second bases might be a vestige of ancient duplication (that shaped the tRNA molecule as a bi-functional adaptor of the genetic code) and in-frame translation of both, sense and antisense, strands of the first protein-encoding genes [12,22,24]. Obviously, the dual complementarity is still preserved by natural selection because of the importance of the second base in coding, in accordance with the principle of evolutionary continuity [55]. Yet, this preservation is not perfect (Table 1), and it is the sub-code for the two mirror tRNA aminoacylations that explains why dual complementarity is still present for certain pairs of complementary anticodons, and not for others. Incidentally, this means that testing the dual complementarity for statistical significance with the 0.5 confidence interval might be too conservative – in some cases dual complementarity might have been lost secondarily, to fit the sub-code of two aminoacylation modes, and, therefore, some sort of conditional/nested probability model should be assumed instead of independence.

In general, the cases of violated or questioned dual complementarity are associated with (1) the cases of mixed-up aaRS class membership and mode of tRNA aminoacylation, i.e. the three aromatic amino acids, Phe, Trp, and Tyr, all presumably of late recruitment [27] and/or (2) the absence of strict complementary partners for the anticodons NNU (this is most pronounced for Thr) (Table 1). If such cases are excluded from the analysis, the dual complementarity is almost perfect (DC = 15/16 = 0.9375), with the Cys(GCA) × Ala(UGC) pair being the only exception, which can be explained by the sub-code for two modes of tRNA aminoacylation.

### The first amino acids could have gained their anticodons before translation

The lack of "evolutionary foresight" necessarily implies that the genetic code, at least in the key features of its "codon-to-aa" assignment (Figure 1), was at first evolving not in anticipation of the catalytic advantage of proteins over RNAs, but simply in response to the current, pressing needs of the RNA world, meaning that the origin of the primary code preceded its use in translation [16,17]. This is certainly true in case of the early ribozyme-mediated operational code – it might not have served originally in translation but rather in replication [47-49], in saving some amino acids from leaking into the environment [33] and primordial catalysis [16,17]. In the latter, Coding Coenzyme Handles (CCH) model, ribozymes use amino acids as cofactors through their direct stereochemical affinity-based interaction with anticodon triplets [16,17]. Consistent with the hypothesis of pre-translation coding, the heptamer and 11-mer palindrome parts of the ancestral acceptor stem have already contained the codon/anticodon-like triplets with adjacent base-determinators. Note in this regard that the debate which of the genetic codes, classic or operational (associated in tRNAs with the anticodon and acceptor stem, respectively), came first is somewhat pointless, as these two codes have diverged from a single ancestor. Not at all pointless, though, is the following question: In which direction did proto-tRNA molecules co-evolve to reach the final cloverleaf shape – from the acceptor (with subsequent gain of the anticodon) or the opposite – from the anticodon (with subsequent gain of the acceptor)? The ancestral palindrome (Figure 5) when considered together with the sub-code for two modes of tRNA aminoacylation and the risk of confusion of complementary anticodons (Figure 3) support the acceptor → anticodon model of tRNA evolution. After all, this model is more consistent with the expansion of the genetic code from the original {G,C} alphabet (to which the repertoire of triplets at 1-2-3 positions was constrained in the double-stranded acceptor) to the final {G,C,A,U} alphabet (possible only in single-stranded antcodons) than the anticodon → acceptor model that implies the reverse, namely {G,C,A,U} shrinking into {G,C} [56]

Care must be taken in the historical reconstruction of the processes that have shaped the code. For example, we do not know the real relevance for the Miller-type experiments for the origin of life (cf. [57]); hence we do not know whether "Miller" amino acids are relevant to the beginnings of genetic code evolution. Also, as the genetic code is likely to have evolved in the context of protocells with complex RNA-catalyzed metabolism [54,58] amino acids produced by such a metabolic network may only partly coincide with the list of prebiotiotically produced ones (by whatever chemical scenario; cf. [59]).

The present paper suggests that the acceptor stem is historically the first, which raises the question about the functional advantage of such an early assignment. Details of this question will be analyzed elsewhere [44], but we tentatively make a suggestion right away. It has been shown by logical arguments that a prolonged coevolution of metabolic networks with membranes is likely to have taken place [60], which makes it likely that selective retention of important metabolites like some amino acids by binding them to, say, RNA molecules was of a selective advantage [33]. It is instructive to look at the first tetrad of amino acids (Gly, Ala, Asp, Val), suggested here, from this point of view. First, some amino acids are needed for nucleotide synthesis, so they are likely to have been important compounds in the RNA world without and before translation or even a catalytic role: Gly and Asp are important here (the glutamine/glutamic acid pair is used for amination only). Another likely constraint is coenzyme synthesis [58]. For example, the biosynthesis of coenzyme A requires Val, Asp and Ala. Further analysis will be provided elsewhere, but this already shows that all members of the first tetrad were metabolically important. It is also logical that a leaky membrane selects first for the selective retention of those amino acids that are most prone to leak out: Gly and Ala from the list.

This suggests a refinement of the coding coenzyme handle (CCH) hypothesis, which can now be broken down into two main steps: (1) Some amino acids (most likely Gly and Ala in the first tetrad) were first linked to short acceptor stem-like structures envisaged in this paper. This must have happened already in a coded fashion. Why? Because it were the other ribozymes that used them for functions (such as nucleotide and coenzyme synthesis). It would have been disadvantageous for the ribo-organisms to have highly ambiguous charging, as already stated in original CCH hypothesis [16]. In this stage the primordial adaptors acted as cofactors exercising group transfer, the groups being amino acids. (2) Presumably recruitment of Asp and the second tetrad (with Arg and His as new members) coincided with the origin of the anticodon loop. It was at this stage that the mechanism of the original CCH hypothesis stepped in: (some) amino acids were now used for catalysis (beyond group transfer: Asp would have had dual functionality), but they were presumably recognized by synthetases and other ribozymes in metabolism via the anticodon loop at this stage.

This revised version of the CCH hypothesis is consistent with all the findings of the present paper. We note that it does not leave much room for classical ambiguity reduction: there would have been selection for reduced ambiguity all along, within the constraints given by an evolving recognition machinery (cf. [61]).

Complementary pairing of nucleotides is probably the most fundamental feature of life and all our results described above indicate that the genetic code itself, as well as the structures and functions of its main adaptors, tRNA and aaRS, have retained imprints of this fundamental complementarity (see also [11-13,22,26-29]). Moreover, approaching the genetic code from this angle might suggest some unexpected novel answers to old, long-standing questions. For example, if the code did predate the translation, then why as triplets? Indeed, in this case we simply cannot invoke any mechanistic or logistic criteria rooted in the optimality of the translation process itself. There must have been something else in having the three-letter coding "words" that provided intrinsic advantage(s) over the two- or four-letter ones. In addition to the already reported advantages [16,17], *we propose here one more: the three-letter "words" in the U, C, A, G alphabet cannot be self-complementary*, whereas the two- and four-letter ones definitely can be. Dinucleotides CG, GC, UA, AU and tetranucleotides CGCG, GCGC, AUAU, CCGG, etc., are perfectly self-complementary palindromes (for example, a complementary partner of CG is, again, CG). We shall discuss the disadvantages of such codes (compared to the triplets) elsewhere. Here we will demonstrate only one of them. According to [41,42], the aa-binding sites of RNA aptamers (selected *in vitro*) contain, more frequently than by chance alone, their cognate triplets, mostly anticodons. One would suppose that putative r-aaRSs could have had such sites. Does it explain why the code is triplet? No, because what really determines the code's "-pletness" is the anticodon of proto-tRNAs and, symmetrically, the anticodon-binding site in r-aaRSs. Consider pairs of r-aaRSs with complementary aa-binding sites that recognized, however, self-complementary anticodons in the duplet or quadruplet codes. These hypothetical codes would pose a much higher risk of confusion and misaminoacylation by such pairs of charging ribozymes (and, later on, enzymes as well) compared to the real, triplet genetic code. (Of course, a quintuplet code, or any other "odd" code for that matter, would also avoid this confusion disadvantage – however, the triplet code is already sufficiently redundant and robust, so any extra complexity would not be of any selective advantage.)

## Conclusion

Thus, although the {G, C} operational and {G, C, A, U} classic codes play a crucial role in translation, their origins from a common ancestor could have been necessitated by the various pressures and challenges of the preceding RNA life. Moreover, the 5'U-XYZ-R3' configuration of the anticodon loop and, accordingly, the sub-code for two complementary modes of tRNA aminoacylation might have originated (and been used by the RNA life) also before translation simply in order to distinguish amino acids with a high catalytic propensity (as ribozyme cofactors) from their complementary partners with a high beta sheet building propensity.

Further investigations, both *in silico *and in the lab, are needed to determine the comprehensiveness of the primary repertoire of proto-anticodons that could have been established before translation. However, it is clear *a priori *that "at some early stage in the evolution of life the direct association of amino acids with polynucleotides, which was later to evolve into the genetic code, must have begun" [62]. In up-to-date terms, the r-aaRSs had to have a site with affinity for a cognate amino acid, perhaps through direct stereochemical key/lock-like binding. Any stereochemical mechanism has less than perfect accuracy due to the finite difference in binding energy between cognate and non-cognate amino acids [16]. On the other hand, it is equally clear that even for the {G,C,A,U} code associated with anticodons, this specificity may have never reached its possible limit. This is because of the editing of aminoacylation, as we know it now (reviewed in [3]). This editing suggests that the hypothetical ribozyme-mediated tRNA aminoacylation may have been, at least for some amino acids, less specific than today – its specificity could not, for example, have exceeded that of the *Aquifex aeolicus *LeuRS with its CP1 domain editing the charged and mischarged noncognate tRNA^Val ^and tRNA^Ile ^[63]. Otherwise, how/why would the r-aaRS → p-aaRS transition need to occur in the first place? Indeed, if it is assumed that the primordial r-aaRS-mediated code had, at some point, achieved the perfect recognition of all 20 amino acids, then it is difficult to imagine the selective advantage(s) of transitioning from these perfectly specific ribozymes to the isofunctional proteins *without editing*. Some sort of co-evolution between the first p-aaRSs and the genetic code shaping must have occurred. And it is during this co-evolution that the code (possibly even expanded compared to its initial complementary core shown in Figure 1B) continued gaining specificity (see also [3,11,22,32]).

## Abbreviations

aa: amino acid; ME: Minimum Evolution; NJ: Neighbor-Joining; p-aaRS: protein aminoacyl-tRNA synthetases; r-aaRS: putative ribozymic precursors of protein aminoacyl-tRNA synthetases; TN: Tamura-Nei (distance); tRNA: transfer RNA; W-C base paring: Watson-Crick complementary pairing, G-C and A-U.

## Competing interests

The authors declare that they have no competing interests.

## Authors' contributions

ASR contributed to the conception of the study and development of the model, carried out the phylogenetic analyses, contributed to the sequence analyses and alignment, and drafted the manuscript. ES contributed to the conception of the study and development of the model, and helped to draft the manuscript. SNR conceived of the study, contributed to the development of the model, contributed to the sequence analyses and alignment, and helped to draft the manuscript. All authors read and approved the final manuscript.

## Reviewers' comments

We are grateful to the reviewers for their thorough and thoughtful analysis and critique of our manuscript. We largely agree with all of the reviewers' comments, and have addressed the corresponding issues either in our response or in the manuscript, when possible (some of the suggestions made by the reviewers actually overlap with our ongoing research in this area – corresponding analyses will appear in the tRNA evolution-themed manuscript that is now in preparation). In our response to the reviewers below we have omitted some minor points brought to our attention (typos, additional references, formatting, terminology, etc.), correcting them directly in the manuscript instead. We have also excluded the (more colloquial) parts of the discussion that were not directly related to the subject of this communication.

### Reviewer's report 1

Rob Knight, University of Colorado, Boulder, CO, USA

#### Reviewer Comments

The origin of the genetic code remains a mystery, despite its centrality to biology. In this manuscript, Rodin et al. reconstruct the ancestral sequence of various tRNA molecules, revealing a palindromic structure in the reconstructed ancestral sequences. They use this model to propose a pathway for the addition of amino acids to the genetic code, linking the model to Szathmary's Coding Coenzyme Hypothesis (the idea that the primordial function of the (anti)codon-amino acid linkage was to deliver amino acids as coenzymes for ribozymes, and that this system was later co-opted for translation).

One problem that should be corrected is the 1980s view of the aminoacyl-tRNA synthetases. We now know that there are more than 20 amino acids incorporated cotranslationally, that there are both class I and class II LysRS activities, many organisms are missing some of the synthetases, etc. If the authors want to argue that the classical picture is ancestral, some data to this effect is really needed. I think it is essential either to recognize the diversity and complexity of the aminoacyl-tRNA synthesis process (as seen in e.g. recent work from the Soll and Ibba labs) or to explicitly make the case that the idealized picture used in this paper really was the ancestral state relevant to the origin of the code. Along these lines, I was also surprised not to see reference to work on the Suga lab on the evolution of tRNA-aminoacylating ribozymes.

#### Authors' Response

We are certainly aware of work from Soll and Ibba labs (as well as Suga's work – see below our reply to the 3^rd ^reviewer). Needless to say, the synthesis of aa-tRNAs (interface between mRNA and proteins) is much more idiosyncratic than we thought it was (in say, early 90s). However, we don't necessarily think that this (presently appreciated) diversity and complexity needs to be contrasted with the classic "idealized" picture of 20 aaRSs evenly divided in two nearly even classes with mutually mirror modes of tRNA recognition. All known deviations from this 10:10 symmetry are subsidiary to the sub-code for two modes of tRNA aminoacylation revealed by us and are partly discussed in previous reports [11,12]. More specifically, these deviations involve both canonical (Cys, Gln, Asn) as well as noncanonical (Sec, Pyl) amino acids that are commonly considered as relative latecomers in translation. Note that their late recruitment is accepted by Soll and Ibba as well (see, for example, [64,65]). Especially telling in this regard is the direct Gln-tRNA formation by GlnRS in eukaryotes versus its indirect synthesis in many bacteria via Glu-tRNA^Gln ^intermediate followed by transamidation routes: remarkably, this difference correlates with the 2^nd ^base of the acceptor stem – absence/presence of dual complementarity in (Leu × Glu) and (Leu × Gln) pairs (Table 1). We will discuss the possible advantages of this difference in a forthcoming report. Here, we would like to note that while stressing the diversity of aa-tRNA synthesis for some amino acids in different kingdoms, Ibba and Soll nevertheless put a strong emphasis on the fact that with respect to tRNA recognition, class I and II aaRSs look like mirror images of each other – the fundamental property that likely reflects their origin (ibid). And, our main ideas and findings are reliant only on just this core complementarity of the genetic code organization.

#### Reviewer Comments

Not enough detail is given for the methods. For example, how was consistency between tree topologies measured, and what threshold was used to determine acceptability? Were the results robust to changes in the reconstruction method and, critically, the substitution model? (One key weakness of most available techniques is that they assume a constant nucleotide substitution process, which we know is not accurate for the diversity of modern genomes due to the heterogeneity of GC contents – software such as Galtier's phylo_win gets around this but may not scale to datasets this large). In cases where multiple tRNAs with the same specificity exist, what decisions were made about which paralogs to align?

#### Authors' Response

We agree that more detailed description of the phylogenetic analysis would be beneficial – we simply thought that much of the target audience might not be interested in technical detail in a first place. Here is a brief summary:

We used primarily three software packages: proprietary software by one of the authors that implements a fast variant of ME method, Mega 4 (NJ and ME) and, coincidentally, Phylo-win . We also used DNAML (from PHYLIP, ) and PAML , but these were very slow, so we did not analyze the datasets in their entirety. The condensed (multifurcations instead of bifurcations for bootstrap values < 50%) trees were largely invariant to the method choice except the bacteria trees that showed variation (d_T _= 4 and 6 for some comparisons). It was the same with respect to the substitution model choice. We went through a full range of appropriate (e.g., not codon-based, etc.) models available, including Galtier and Gouy, and only condensed bacteria tree showed variation (d_T _= 2, 4 and 6 for some comparisons). Importantly, this did not change our main conclusions (see below).

For the actual trees to be included in the manuscript, we selected Mega 4 NJ with Tamura-Nei distances for the three reasons: first, Mega NJ implementation is a popular and convenient one, thus enabling the readers to easily duplicate or (built upon) our results; second, TN distance is a robust compromise between too-simple on one side and possibly overfitting on the other side models; third, the topologies were consistent with the ones obtained in previous studies using different (genomic, etc.) methods (see, for example, [21]).

In general, it is unclear which method/model is "the best" for tRNA phylogenetic analysis. In our forthcoming (and substantially more technical) manuscript on tRNA evolution we will, among other things, touch on this issue.

About the paralogs: some of these cases might have represented pseudogenes. There were no strict rules for choosing "true" paralog(s). However, all other things being equal, the likely pseudogenes (with strongly distorted secondary structure) were manually removed (thus not contributing to the phylogenetic tree reconstructions and DC calculations).

#### Reviewer Comments

What criteria were used in the manual assignment of ancestral states, and how did this manual assignment compare to model-based approaches such as likelihood and Bayesian approaches? (These are much slower, and might not be feasible for the full dataset, but could certainly be used to validate the manual methodology on a subset.) The claims made in the paper about the conservation of very deep palindromic structure are surprising, and the reader needs to be reassured that the results are robust to the many choices that can be made in this type of phylogenetic analysis.

#### Authors' Response

This is a very good point and we actually did validate our manual methodology by applying PAML to the subset of the Eukarya dataset. (We plan to apply model-based approaches, Bayesian and likelihood-based, to the datasets in their entirety in the future – time, computational resources and scalability permitting). Our manual reconstruction relied on simple Fitch's parsimony-based method [23] and can be essentially summarized as a "common sense" union/intersection algorithm. In addition, one could also construct straightforward consensus sequences, but these would be more biased than the ancestral sequences due to the fact that some subgroups of species are relatively over- or underrepresented in the tRNA database. Therefore, for the purposes of this report manually reconstructed ancestral sequences made the most sense. As far as "very deep palindromic structure" is concerned, our analysis reveals that the CCR motif at 5-6-7 positions is very robust. However, while the CCR motif is virtually "set in stone" in Archaea and Eukarya, it is less robust in Bacteria – in the 5^th ^position (for averaged isoacceptor tRNAs of 20 amino acids) we observe 10C to 8G (with ambiguous 2S).

#### Reviewer Comments

I am concerned about the use of the binomial distribution to assess statistical significance of the DC as it makes the assumption that each pair of tRNAs is independent, yet the same tRNA sequence contributes to many pairs. It would be useful to check whether this violation of assumptions matters by a Monte Carlo approach to derive a null distribution empirically (in our experience, this type of model violation can result in orders of magnitude difference in apparent statistical significance in other problems: it might not be important in this situation, but then again, it might be). I would also argue that stratifying by domains could be useful as you could then treat each domain as an independent test of the hypothesis and use Fisher's method to combine the probabilities (assuming that there has been no horizontal transfer of tRNAs and that the tRNAs in each domain for a given specificity are monophyletic – on the other hand, you could certainly make the case that these assumptions are not necessarily warranted).

#### Authors' Response

We completely agree, and the work is actually underway on deriving the empirical null. This will be one of the focal points of our tRNA evolution manuscript that is now in preparation (another point is discussed below). This approach is both technically and computationally involved, however, and at this time we decided simply to give the readers the actual "bin counts" so that the readers can decide for themselves – but we agree that binomial-derived p-values might be oversimplified.

The idea to combine stratified test results using Fisher's method is a good one and, frankly, did not occur to us. However, we are almost sure that the assumptions will be violated. For example, one can see the significant dual complementarity separately for NGN × NCN and NAN × NUN pairs in domains of eukaryotes and eubacteria, but only for NAN × NUN pairs (and not for NGN × NCN pairs) in archeabacteria (Table 1). We are unable at the moment to pin down the possible reason(s), although our preliminary observations point to a possibility of lateral tRNA gene transfer as an interfering factor. Again, this is something that we are working on right now as a part of our methodological tRNA evolution study.

#### Reviewer Comments

The pathway for code expansion proposed is consistent with the data presented, but I am unsure that it is uniquely consistent. In general, there are so many pathways for code expansion that have been proposed, all of which are plausible within their own framework but all of which disagree with one another to a greater or lesser extent except for a few basic assumptions that may stem from intuition and hence publication bias (e.g. the small amino acids came earlier) that it is difficult to find this particular pathway more convincing than the others. Some sort of empirical test that would discriminate amongst the various possibilities is really needed, but beyond the scope of this paper.

#### Authors' Response

What the reviewer states here is undisputable. Evolutionary biology research is singular in that its results and hypotheses are often impossible to validate. This is especially true for the early (and thus ancient) evolutionary events, such as the genetic code origin, and is in sharp contrast to the "standard" biomedical/genetic research paradigm, in which validation is essential. Indeed, consider a study (fairly representative of the contemporary biomedical/genetic research in general) where one aims to find a gene influencing a certain human trait of interest via either hypothesis-driven (e.g. candidate gene) or data-driven (e.g. genome-wide association) approach. Once a gene is identified, one can validate it epidemiologically (by replicating the association in a large independent sample) or directly, by knocking out, rescuing, expressing, etc., this gene in various animal models. This, obviously, cannot be done with the genetic code origin research. Instead, we must rely on indirect empirical tests (indeed, beyond the scope of this paper), model selection/fit/averaging principles (Occam's razor, likelihood, etc.), and plain common sense.

One of the authors is also interested in reconstructing Bayesian networks from genetic epidemiology data – and no matter how dimensionally "favorable" (few variables, many observations) the dataset is, the robust reconstruction of the one "true" network is unlikely. However, many robust *features* can be reliably inferred (using, for example, bootstrap, just like in phylogenetics). Same with the code origin scenarios: it would be presumptuous to state "this is the only pathway!" However, some robust features, common to various theories and pathways, do exist. In our opinion, one such feature is the combination of the dual complementarity, the sub-code for two modes of tRNA recognition by present-day aaRSs (and presumably by their ribozymic isofunctional precursors) and the hypothesis of in-frame usage of both complementary strands of primordial genes [30-32] – all these complementarity-based findings are consistent with each other, and can hardly be explained by chance alone (especially the latter).

#### Reviewer Comments

It's not clear to me why catalytic propensity would leap out as the explanation for separating the lists of amino acids (His, Asp, Glu, Lys, Arg) and (Val, Ile, Leu, Phe, Ala) rather than, say, hydrophobicity (which is known to play a huge role in protein structure and is the one property that invariably comes up as important in studies of code optimization.

#### Authors' Response

First, as recognized (arguably somewhat late) by Kun et al. [39], the coding coenzyme handle hypothesis generates the prediction that catalytic propensities of amino acids should not be distributed randomly in the genetic code, and it is reassuring to see that this is indeed the case. Second, this automatically clusters catalytically unimportant amino acids as well. The remarkable thing is that these amino acids belonging to these two clusters have complementary anticodons. Third, the catalytically unimportant amino acids turn out to be important in building scaffolds. Fourth, as enzymes are essentially catalytic amino acids held in position by scaffolds, there seems to be a seamless way to enter the protein world, by venturing into the complementary worlds of structurally and catalytically important amino acids at the same time, enforced by dual complementarity. Fifth, although it is true that catalytically important amino acids are hydrophilic, this correlation is of course not surprising. As the Reviewer observes, the question is which trait is primary. IF we accept the idea that coded amino acids preceded translation and long peptides, we believe the choice of catalysis over hydrophilicity is more compelling because of the clear nature of the selective forces involved.

#### Reviewer Comments

In general, I think the discussion could benefit from a rewrite: the explanations given are typically plausible and consistent with the story, but also consistent with many other interpretations.

Overall, my opinion is that this manuscript provides an interesting speculation on the origin and evolution of the genetic code rather than the last word. However, the ideas it presents are certainly worth discussion and thus worth publishing, especially once the issues noted above are addressed.

#### Authors' Response

We definitely agree that this is not the "last/only" word. We hope we made it more unambiguous in the discussion above.

### Reviewer's report 2

Juergen Brosius, University of Muenster, Muenster, Germany

#### Reviewer Comments

It is highly conceivable that, for functional reasons, RNA stems were aminoacylated long before the advent of templated protein biosynthesis. Even simple aminoacylated stems in close proximity to each other could have been involved in spontaneous peptide bond formation. Other RNAs (a primordial ribosome) could have facilitated the reaction (perhaps simply by bringing aminoacylated stems into the required close proximity). This possibly set the stage for the advent for templated peptide/protein biosynthesis and at this juncture, it is thought that the primordial tRNA stem with the operation code (the "charging code") duplicated leading to the code, mirrored by the anticodon in tRNAs. The authors argue that a single base (tRNA position 2) and its environment point to such an ancestry. Furthermore, the different modes of aminoacylation on different faces (minor versus major groove) of the aminoacyl stem of tRNAs with class I and class II aaRSs and their presumed ribozyme functional predecessors, r-aaRSs, might have had their origin in the avoidance of mis-aminoacylation when complementary "codons" of the "operational or charging code are involved.

#### Authors' Response

Not exactly so. Rules of the sub-code for tRNA recognition by r-aaRSs are formulated for pairs of complementary anticodons, and not for their possible double-stranded precursors in the acceptor stem (even though different faces of the acceptor, major *vs*. minor groove, do define two sterically mirror modes of aminoacylation). What the sub-code, in fact, does, it minimizes the risk of confusion for complementarily encoded amino acids. However, it turns out that for precisely the presumably earliest pairs of such amino acids (Gly – Ala, Gly – Ser) this risk does not depend much (if at all) on the anticodon loop. In contrast, the 2^nd ^base pair of their acceptor stems does matter here. Moreover, a closer inspection suggests that the major groove side of tRNA recognition corresponds to the primordial operational (charging) code, whereas the minor-groove-side was brought into action somewhat later, after the tRNA molecule has gained the anticodon loop.

#### Reviewer Comments

While the data are compelling and the underlying hypothesis well worth entertaining, there are a number of problems associated with this proposal:

1) Aminocylation of a primordial tRNA with a r-aaRSs in trans with a reasonable amount of specificity would have involved some complementarity between those two RNA molecules and the acceptor "stem" should have had a different structures with at least a bulged operational code area.

#### Authors' Response

**Figure 7:**
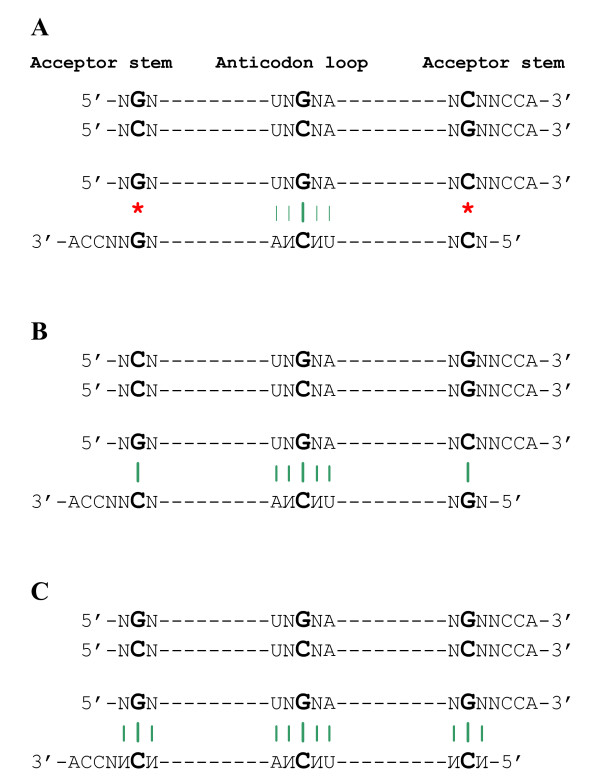
**The dual complemenarity might provide indirect evidence in support of the existence of small local "bulges" in the earliest precursors of the acceptor stem**. (A) Shown is a pair of proto-tRNAs with complementary anticodons that has, in parallel, complementary 2^nd ^bases in their acceptor stems. When aligned head-to-tail, these sequences reveal mismatches at the 2^nd ^position (at least), meaning that the two could not have originated concertedly (as a pair) with one proto-tRNA being a complementary replica of another [26,28,29]. (B) The 2^nd ^base pairs are identical in these two tRNAS with complementary anticodons meaning that, in contrast to "A", their concerted origin is possible, but the dual complementarity is not. We have noted this difference between A and B before, but interpreted it in a different way 2[6]. (C) In this case, proto-tRNAs with complementary anticodons could originate concertedly (as a pair of + and - sequences) but, in contrast to "B", their 2^nd ^bases in the acceptors are also complementary. However, simultaneous maintenance of these two properties is possible if and only if there are local distortions of acceptor helices ("bulges") just at the 2^nd ^position. Remarkably, such local mismatches could facilitate recognition of anticodon precursors adjacent to the universal NCCA 3' terminus by putative ribozymes with aminoacylating activity. The fact that the dual complementarity does exist (Table 1) favors this ("C") case.

This is a real problem and, obviously, solution(s) is(are) to be found in the expansion of the tRNA molecule from a primordial helix (Figure 5) to the extant cloverleaf. Supplemental figure 5 shows one of the models of this expansion that in fact combines (and is partly consistent with) those of Bloch et al [66] and Di Giulio [15]. Other possible models of this expansion process will be presented in our forthcoming paper. Here, we would like to note the following:

First, it was emphasized in our recent paper ([12], p.351) that *"Watson-Crick pairing-based recognition of the operational proto-code by r-aaRSs might imply a local distortion of the acceptor helix. Interestingly, interactions of typical class I protein aaRSs with tRNAs do cause serious changes of the acceptor stem end, including unwinding and disruption of base pairing"*. Second, the signature of the acceptor arm is the unpaired 5'NCCA3' tetramer, and it appears that the reconstructed ancient heptamer might have already contained it (Figure 5). That is, before the formation of the proto-acceptor helix, its single-stranded 5' half-precursor could have been easily recognized by r-aaRSs *via *the usual W-C pairing and more or less specifically charged at CCA3' terminus. Third, it is clear that as soon as the double-stranded acceptor stem was formed, the primordial charging code did require a local bulge-like distortion of the stem, in order to (at least) maintain this recognition by r-aaRSs. Remarkably, the dual complementarity might point to the existence of such ancestral local bulges. Figure 7 shows why and how. If, in accordance with [26-29], proto-tRNAs with complementary anticodons originated concertedly as pairs of complementary (+ and -) sequences, then the dual complementarity would be observed if and only if the stem is distorted just at the 2^nd ^position (at least). Later, after the charging proteins supplanted their ribozymic precursors, such mispairings were no longer required.

**Figure 8:**
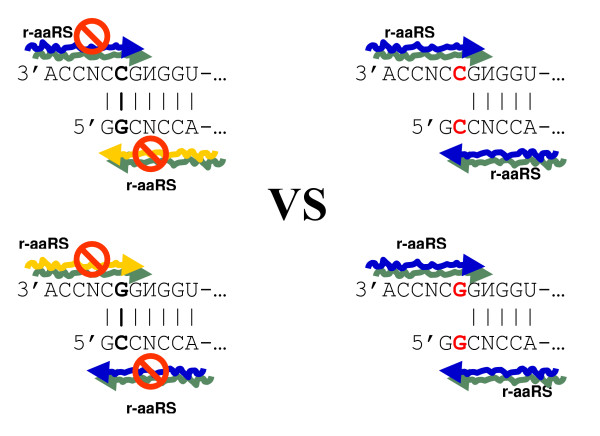
**Bulges at the second position of the acceptor stem might have been a factor in minimizing the risk of confusion of earliest adaptors for complementarily encoded amino acids (see text for details)**.

Furthermore, the most frequent at 1-2-3 positions of the acceptor stem are complementary 5'GGC3' and 5'GCC3' triplets. Figure 8 shows common ancestors for the corresponding acceptor arms (3' strand of each of them is a perfect palindrome). What we have overlooked before is that the dual complementarity (still detectable) suggests that this pair of ancestors might have occurred in two versions. In case of the variant shown on the left, the stem structure is perfect, i.e., contains no bulges. However, because of the palindrome-associated symmetry, there is a high risk of confusion of these two acceptors if the cognate r-aaRSs recognize them from the opposite sides. Accordingly, we claimed the same sides as more suitable for recognition by r-aaRSs (see text). In turn, of the two sides, the major groove one appears to be preferable simply because it contains the unpaired NCCA3' terminus. However, again, just because of the palindromic structure, r-aaRS of one acceptor has an increased risk to recognize its complementary partner from the opposite site (see text for detail). And it is probably for this reason that of all the pairs of tRNAs with complementary anticodons none has GGC and GCC triplets at the first three positions of their acceptors stems.

At any rate, any pair of proto-acceptors with GGC and GCC was still confusion-prone for putative r-aaRSs (see Figure 6).

Shown on the right (Figure 8) is another version, the one that accords fully with the origin of proto-tRNAs as complementary images of each other [26,28,29]. This version has mismatches C*C and G*G at the second position in the acceptor helices. When compared to the left version, the right version reveals a number of advantages. First, in each of the two acceptors, major and minor groove sides have precisely identical sequences for recognition by r-aaRSs, but if even the sides were confused, it would not matter in this case (in contrast to the left case). Second, there is no way of confusing the two acceptors under recognition by r-aaRS from complementary partners. Third, due to this mismatch at the 2nd position, the major groove side is apparently more exposed to r-aaRS (for recognition) than minor groove side (which would not be the case for the perfect acceptor stems in the left version).

Finally, acceptors with mismatches are generated by duplications of the original palindrome (Figure 7). We will consider this and some other models of tRNA growth (from a proto-anticodon and NCCA to the complete 76 base-long molecules) in our next paper. Here we would like to note that: 1) duplications of the palindrome on one (+) strand are automatically accompanied by duplications of its mirror complementary image on the opposite (-) strand, 2) this is one of the very few (at least to our knowledge) ways by which different small precursors of tRNAs (making up a primary genetic code, perhaps even before translation) could in parallel, cohesively, reach the final cloverleaf shape, and 3) this is precisely the way that produces two acceptor stems with bulges at the crucial location.

Thus, of the two variants shown in Figure 8, the one on the right, with G*G and C*C mismatches at the second position, appears to be better protected from mis-aminoacylation. Yet, we are unable to choose between the two because if for the early, complementarily encoded (even before translation) amino acids their proto-adaptors did have these bulges, the latter were likely to proffer a very short-term "advantage". At any rate, cases of such mismatches in the tRNA database [18] are too infrequent for true "living fossils". Most importantly, no matter which variant is in fact a true one, the very first adaptors for the complementarily encoded amino acids were recognized and distinguished by r-aaRSs *via *their acceptor stem, not the anticodon loop – therefore, likely even before the advent of translation.

#### Reviewer Comments

2) If the anticodon stem/loop and acceptor stem are homologous (in the true sense of having common ancestry instead of merely sequence or structural similarity), then the two codes a) shifted on the structure and b) became double stranded in the acceptor stem, whereby the proposed identity of bases in the acceptor stem with the anticodon is much more difficult to reconcile.

#### Authors' Response

We do not really see the difficulty here because the two codes, charging and classic, both have been initially quite ambiguous. Among the evidence in support of such ambiguity especially telling is the fact that the concerted complementarity of 2^nd ^bases in the acceptor is observed for pairs of tRNAs with completely complementary anticodons, but is not observed for pairs in which only the central bases are complementary [22]. Also supportive (though indirectly) is the fact that for the presumably early amino acids, Gly, Ala, Ser, etc. (see quarter I in Figure 3), there were no reports (to the best of our knowledge) on successful selection of RNA aptamers (see, for example, recent reports from Yarus group) with significantly increased content of cognate triplets in amino acid-binding sites. Actually, if the sub-code concept (Figures 1B and 3) holds, we should not expect to see such reports at all [12]. Clearly, ambiguity of the primordial charging code in the proto-acceptor has been reduced during the r-aaRS-mediated expansion of the classic code in the anticodon area (quarters II and III in Figure 3). The final shaping of the code was likely taking place under control of the protein synthetases. But the latter are not necessarily needed (or at least are not as much needed as the ribozymes) to provide aa-specific recognition of tRNAs in the bulged bases of the acceptor helix and/or in single-stranded anticodon loop. Experiments by Schimmel group [2-4] made it unequivocally clear.

#### Reviewer Comments

3) Should complementarity of the primordial tRNA and the r-aaRS not have played a role in this interaction in trans, of what nature was the RNA-RNA interaction? Some base fingers in the respective grooves might be possible but without other interactions it might be hard to resolve the problem of specificity. Perhaps the ribosomal RNA structure with tRNAs bound might give us some insight. Perhaps, this is the solution to the question of what was the ancestor of ribosomal RNA. In the past, I entertained RNA molecules akin to tmRNA, that could act both like a tRNA and a mRNA [67]. Could r-aaRS have been the ancestor of the primordial ribosome?

4) Back to the problem of aminoacylation: Perhaps, specificity was achieved by aminoacylation reaction in *cis *(that is, tRNA was a self-aminoacylating ribozyme) which would have rendered unnecessary the base-pairing (or other specific interaction) between two separate RNA molecules. In this case too, the molecule could have been a rRNA ancestor.

#### Authors' Response

These two questions are interrelated. The self-aminoacylating tRNA as an ancestor of rRNA is an interesting hypothesis, feasible for verification. The cis- and trans-possibilities are also worthy of attention. In particular, consistent with the idea of proto-tRNA as a self-aminoacylating ribozyme is the remarkable positioning of the introns in tRNA genes, usually after 37^th ^nucleotide, just where the putative r-aaRSs could have resided ([53], see also[16,17]). In addition, as we have mentioned before in [56], if selective complexes of the C4N type proposed in [68], with even a weak stereochemical affinity between amino acids and anticodon-like triplets, did have occurred at 1-2-3 positions of proto-acceptors, then neither cis- nor trans-acting amino acid-specific ribozymic predecessors of aaRS were necessary for the code evolution to get started – all the specificity required was provided by the primordial charging code in the proto-acceptor. The dual complementarity basically supports this idea. Furthermore, the perfect complementary symmetry of tRNA recognition by putative r-aaRSs points to the possibility that one such tRNA could have originally catalyzed the aminoacylation of its complementary partner and *vice versa *[56]. Thus, something like trans-"self"-aminoacylation could have taken place at the very beginning.

At any rate, postulation of the ancient more or less specific aa-RNA complex is unavoidable, never mind whether it was a proto-tRNA itself or r-aaRS, and whether it provided this specific charging in cis- or trans-position. More importantly, while not denying any and all of the above possibilities, we cannot yet imagine how the sub-code for two modes of tRNA aminoacylation, dealing with complementary anticodon loops, could emerge without W-C pairing-based recognition by putative ribozymes. It should also be noted that in the model of ribosomal evolution [67] it remains unclear where the single-stranded anticodon came from, whereas this is one of the central aspects of this current study.

#### Reviewer Comments

"The complementary pair-based scenario of early code shaping renewed the interest in the division of p-aaRSs into two classes, and suggested they originated from the complementary strands of the same ancestral gene [30-32]." I followed these ideas 1.5 decades ago, when it was suggested that genes encoding neuropeptide precursors and their receptors were originally encoded on opposite strands, ideas originally forwarded by J.E. Blalock. Not much came out of it and I think they are far fetched. In the simplest cases then, poly-Lysine should bind poly-Phenylalanine and poly-Proline should bind poly-Glycine or even the grooves of polyU/poly(A) or poly(G)/poly(C) RNA duplexes. One can always detect artifactual peptide to protein or protein to RNA binding – if one wishes so.

#### Authors' Response

Our idea of the origin of two p-aaRS classes from complementary strands of one ancestral gene is based on the logic premises and data [22,30] that have nothing to do with the theory of mutual binding affinity of complementarily encoded proteins. By the way, to the best of our knowledge, this theory was actually put forward as early as in 1969 [69]. While we do not necessarily share the authors' enthusiasm for this code-based hydropathic anti-complementarity of amino acids, we would like to stress once more that the ancient RNA world was likely much more strand-symmetric with regard to the emerging code and translation, so that both (+ and -) complementary replicas of genes could have been used as mRNAs. In fact, it is precisely the continuing evolution and specialization of the genetic code and translation that, among other factors, made current "bilingual" life so much strand-asymmetrical.

#### Reviewer Comments

"Therefore, it appears that the leading role in maintaining the specificity of tRNA aminoacylation must, at some point, have passed from the acceptor stem to the anticodon and then, after the genetic code established its complementary core, must have returned back to the acceptor stem (accompanied by the r-aaRS → p-aaRS transition)..." and suddenly covered shorter or larger distances. First, a short distance, then, after duplication, a large distance and then a short distance again to the CCA end. This is confusing.

#### Authors' Response

In our opinion, there is no confusion here. These "forth and back" passages of tRNA recognition reflect main steps in evolution of the code mediated initially by aminoacylating ribozymes and then by their protein successors. Undoubtedly, first protein p-aaRSs (of both sterically mirror types) could have appeared, and supplanted the isofunctional ribozymes r-aaRS, only when the latter have already shaped the complementary core of the genetic code (Figure 1B). This shaping minimized errors in recognizing and distinguishing just complementary anticodons. Also without a doubt, these first p-aaRSs were simply too small to cover both major coding sites of tRNAs – one (charging code) in the acceptor and another (classic code) associated with the anticodon. However, at that stage there was no need for such "prolonged" recognition – one code site was physically associated with another one within the same tRNA molecule. The primordial charging code, located immediately next to the amino acid attachment site, was obviously a better candidate for the further tune-up during co-evolution with anticodons under the control of first small p-aaRSs. Work by Schimmel group demonstrates that it is still the case for ten amino acids (see [3] for review). For other ten, in order to avoid mis-aminoacylation, p-aaRSs (as r-aaRSs before) had to spread the tRNA recognition by adding (quite idiosyncratically) the anticodon-binding domain. Thus, the acceptor-to-anticodon transition under p-aaRS control recapitulates a similar transition under r-aaRS control (but with the complementary core of the genetic code already established).

#### Reviewer Comments

Who can be sure that genomes in the RNA or RNP worlds were double stranded?

#### Authors' Response

Replication of any genome, no matter single- or double-stranded, necessarily passes through building a complementary copy. Single-stranded plus RNA sequences replicate through their minus replicas. We simply meant that at the origin of the genetic code and translation both, + and -, strands were not likely to be originally differentiated into sense and anti-sense ones.

#### Reviewer Comments

The authors could possibly discuss [70] as well as [8].

#### Authors' Response

We intend to do just that in our forthcoming manuscript, currently in preparation (it would be a better fit, thematically).

#### Reviewer Comments

"Indeed, if it is assumed that the primordial r-aaRS-mediated code had, at some point, achieved the perfect recognition of all 20 amino acids, then it is difficult to imagine the selective advantage(s) of transitioning from these perfectly specific ribozymes to the isofunctional proteins *without editing*." How about speed?

#### Authors' Response

Of course, speed could have been one of the advantages of r-aaRS → p-aaRS transition, even though aminoacylation by p-aaRS remains the slowest stage in modern translation. However, the first proteins encoded *via *r-aaRSs have included... p-aaRSs. Replacement of these very first p-aaRSs synthesized with the help of slow but specific r-aaRSs by their analogs synthesized with the help of rapid but less specific p-aaRSs (themselves) is actually similar to mutations with pleiotropic effects which, in this particular case (synthetases) would lead to the error catastrophe [1].

#### Reviewer Comments

"This means that in the very beginning of development of the code, the choice between the two complementary modes of tRNA recognition did not depend much, if at all, on the anticodon loop." – if there even was one at that stage!

#### Authors' Response

We are not sure that we understand the reviewer correctly... If we do, it should be noted that, actually, it was this anticodon-independence that (among other indirect arguments) led us to the hypothesis that operational (charging) code embodied mostly in the acceptor preceded the classic code embodied in the anticodon.

#### Reviewer Comments

"It was obvious that the simplest solution of the entire problem would be to have an anticodon/codon homolog in the acceptor stem..." The authors should be cautious and only use the terms homolog/homology if common ancestry is really proven. Otherwise the terms (sequence) similarity or complementarity, where applicable, should be used.

#### Authors' Response

The reviewer is correct. Our "would be" was supposed to indicate that what we were talking about was a hypothesis.

#### Reviewer Comments

"Recognition of anticodons by r-aaRS ribozymes was likely based on the complementary base pairing [16]." I thought it was always the recognition of operational codes on the acceptor stem.

#### Authors' Response

No, and this misperception is important. Therefore, we clarified the text and put a strong emphasis on the fact that we estimated the risk of confusion for single-stranded complementary anticodon loop (see Figure 3), not for their hypothetical precursors in the acceptor stem.

### Reviewer's report 3

Anthony Poole, Stockholm University, Stockholm, Sweden

#### Reviewer Comments

This is a fascinating paper that builds on the recent comprehensive review by Rodin & Rodin in Heredity (2008; vol. 100: 341–355). The key idea presented here by Rodin, Szathmáry & Rodin is that the genetic code (i.e. that associated with codons in the genetic material, and anticodons on tRNA) and the operational code (whereby tRNAs are correctly charged by aminoacyl-tRNA synthetases) share a single origin. Furthermore, the authors argue that on current data it seems reasonable to conclude that aminoacylation predated cooption of primordial 'tRNAs' into translation.

For readers who have not already read the Heredity paper, I can recommend reading that first because much of the material in the early part of the current paper is covered in greater detail there. There is some overlap, but on balance I think this is necessary so as to adequately introduce key background concepts, along with several important recent developments; unfortunately, to permit the authors to adequately focus on the newer ideas presented here, some of the review material from the Heredity article is covered in a condensed form, which may make the paper difficult to follow for those who do not already know the earlier body of work. Still, I think this is more a consequence of the complexity and size of the topic being addressed than it is some shortcoming of the current paper.

Overall, I think the ideas presented here are plausible, and extremely interesting, and the authors indicate a productive path that may be taken in order to arrive at a clearer understanding of the origins of the genetic code. The argument that the two codes originated as a single code is elegant, and the available data do seem to support this contention. One interesting aspect is the further development of the coding coenzyme handle hypothesis within the context of the RNA operational code. In particular, the order in which amino acids seem to have been coopted into the modern code strengthens the idea that the earliest usage of amino acids attached to RNA was in biosynthetic and catalytic roles. A number of authors have previously argued that an origin for the ribosome and tRNAs in replication seems likely. Considering briefly the genomic tag hypothesis in the context of the current work, perhaps one shortcoming of that model is that it is not clear a broad repertoire of charged tRNAs need evolve, nor is an obvious explanation forthcoming concerning the stepwise addition of amino acids to the genetic code. In contrast, the biosynthetic/coenzyme view espoused by Rodin, Szathmáry & Rodin does provide that perspective, and is a welcome development; the model presented here is on far firmer ground than the convenient ordering of extant examples of viral replication into a progression in an attempt to account for the evolution of tRNA, as per the genomic tag hypothesis. Having said that, I don't see an obvious conflict between the idea that the decoding function of the ribosome was initially involved in replication, nor that charged 'tRNAs' could have been coopted into replication at some point in the evolution of this process.

One matter I wanted to briefly comment on regards the possibility that the first genetically-encoded proteins may have been RNA 'chaperones' [71]. In this model, exact sequences would have been less important than general attributes such as positive charge (thereby permitting binding to and stabilisation of existing ribozymes or functional RNAs). One aspect of that idea that bugged me was that amino acids such as arginine, which is prominent in a wide range of RNA-binding motifs, and glutamine, which can improve binding affinity (see [70]) are not among the amino acids consistently argued to be the earliest additions to the genetic code. Yet the emergence of low complexity peptides where exact sequence order would not be crucial to function seems far more plausible than the old idea that the first proteins would have been catalysts. Rodin, Szathmáry & Rodin proposal that the acceptor stem likely predates the anticodon loop, together with the recognition that the two codes could have a single origin, is interesting in this context in that it seems likely that a significant repertoire of charged tRNAs would have predated protein synthesis, so my impression is that there really is no conflict between code evolution and an RNA chaperone function for the earliest genetically-encoded proteins; these may have been largely separate and independent events.

#### Authors' Response

We share the reviewer's opinion that the earliest, pre-translation, acceptor stem – associated genetic code and the RNA chaperone function of first encoded proteins do not actually contradict each other; on the contrary, the latter rather complements the former when we think of the RNA → RNP transition in general and the r-aaRS → p-aaRS transition in particular. More specifically, according to [73,74], two "minimalist" complementary p-aaRSs from the opposite classes might have simultaneously made 'contact (from major and minor groove sides) with one tRNA acceptor stem, thus covering and protecting it like "chaperons". This chaperoning turned out to be quite selective, consistent with the separation of each p-aaRS class into three subclasses (Ia, Ib, Ic and IIa, IIb, IIc, respectively) – Ia synthetases can be paired with IIa, Ib with IIb, and Ic with IIc, whereas binding of two p-aaRSs from different subclasses to one acceptor is sterically forbidden (ibid). Remarkably, in full accord with this selectivity, the ancestral acceptors more often than not have the same 2^nd ^base pair for the sterically compatible synthetases and different 2^nd ^base pairs for the sterically incompatible ones. Certain methanogenic archaeae give us a unique practical demonstration of this possibility: a LysRS-like protein (pLysRS), a homolog of subclass Ib LysRS that activates the noncanonical amino acid pyrrolysine, together with the regular subclass IIb LysRS can indeed bind simultaneously to the same tRNA^Lys ^([75], see also [3]).

#### Reviewer Comments

A minor fact let for the discussion on page 12: in vitro RNA selection experiments have been published that indicate ribozymes can perform aminoacylation (Lee, N., Bessho, Y., Wei, K., Szostak, J.W., and Suga, H. 2000. Ribozyme-catalyzed tRNA aminoacylation. *Nat. Struct. Biol*. 7: 28–33.)

#### Authors' Response

We have referred to this paper in our original communication on the sub-code for two modes of tRNA aminoacylation [11]. However, this is a very important work, indeed (see also the first review), and therefore we made the corresponding correction in this paper as well (see ref. [36]).

#### Reviewer Comments

Finally, I did raise an eyebrow at the use of ancestral sequence reconstruction in this work, but I don't think it's crucial to the result reported here; it seems that there is substantial overlap between consensus sequences and the results from ancestral sequence reconstruction, and as the aim is to try and extract some indication of the presence of a functionally-important motif at the acceptor end of the tRNAs, I think the approach used is probably sufficient for the stated aim.

It may be useful to provide more detail on the ancestral sequence reconstruction procedure – for instance, stating that the trees were rooted (see Additional files 1, 2, 3 and 4). Furthermore, it wasn't clear to me whether the manual reconstruction was solely using parsimony. I presume this was the case, but it would help to be explicit.

#### Authors' Response

These are two very good points, also made by the first reviewer. Accordingly, please see the detailed discussion above.

## Supplementary Material

Additional file 1 **Supplemental figure one**. Archaea phylogenetic tree. *Halobacterium sp*. and *Methanosarcina mazei *were used as a composite internal outgroup to root the tree. Bootstrap % values are based on 10,000 replications. Click here for file

Additional file 2 **Supplemental figure two.** Bacteria phylogenetic tree. *Halobacterium sp*. and *Methanothermobacter thermautotrophicus *were used as a composite external outgroup to root the tree. Bootstrap % values are based on 10,000 replications. (The 35 species used to reconstruct the tree are the same used by Wolf et al in [21]. The full tree incorporating the sequences from 132 Bacteria species present in [18] is available from the authors upon request). Click here for file

Additional file 3 **Supplemental figure three.** "Condensed" Bacteria phylogenetic tree. The internal tree branches not supported by bootstrap (i.e. with the bootstrap values less than 50%) were collapsed, resulting in multifurcations. This "condensed" tree topology was robust with respect to the phylogenetic reconstruction method and substitution model. Click here for file

Additional file 4 **Supplemental figure four**. Eukarya phylogenetic tree. *Halobacterium sp*. and *Methanothermobacter thermautotrophicus *were used as a composite external outgroup to root the tree. Bootstrap % values are based on 10,000 replications. Click here for file

Additional file 5 **Supplemental figure five.** One of the plausible models of elongation of the ancestral palindrome (see Fig. 5 in the text) to the tRNA cloverleaf. The model is based on duplications by self-priming and self-templating. The final cloverleaf has many of the sites of splitting tRNAs on minigenes in archaeal parasite Nanoarchaeum equitance 50 51, as well as the positions of processing in permuted tRNA genes from red algae Cyanidioschyzon merolae 52. Click here for file
