# Supplementary figures and images for: One ancestor for two codes viewed from the perspective of two complementary modes of tRNA aminoacylation
Source: Biol Direct. 2009 Jan 27;4:4. doi: 10.1186/1745-6150-4-4 (PMC2669802; doi:10.1186/1745-6150-4-4)

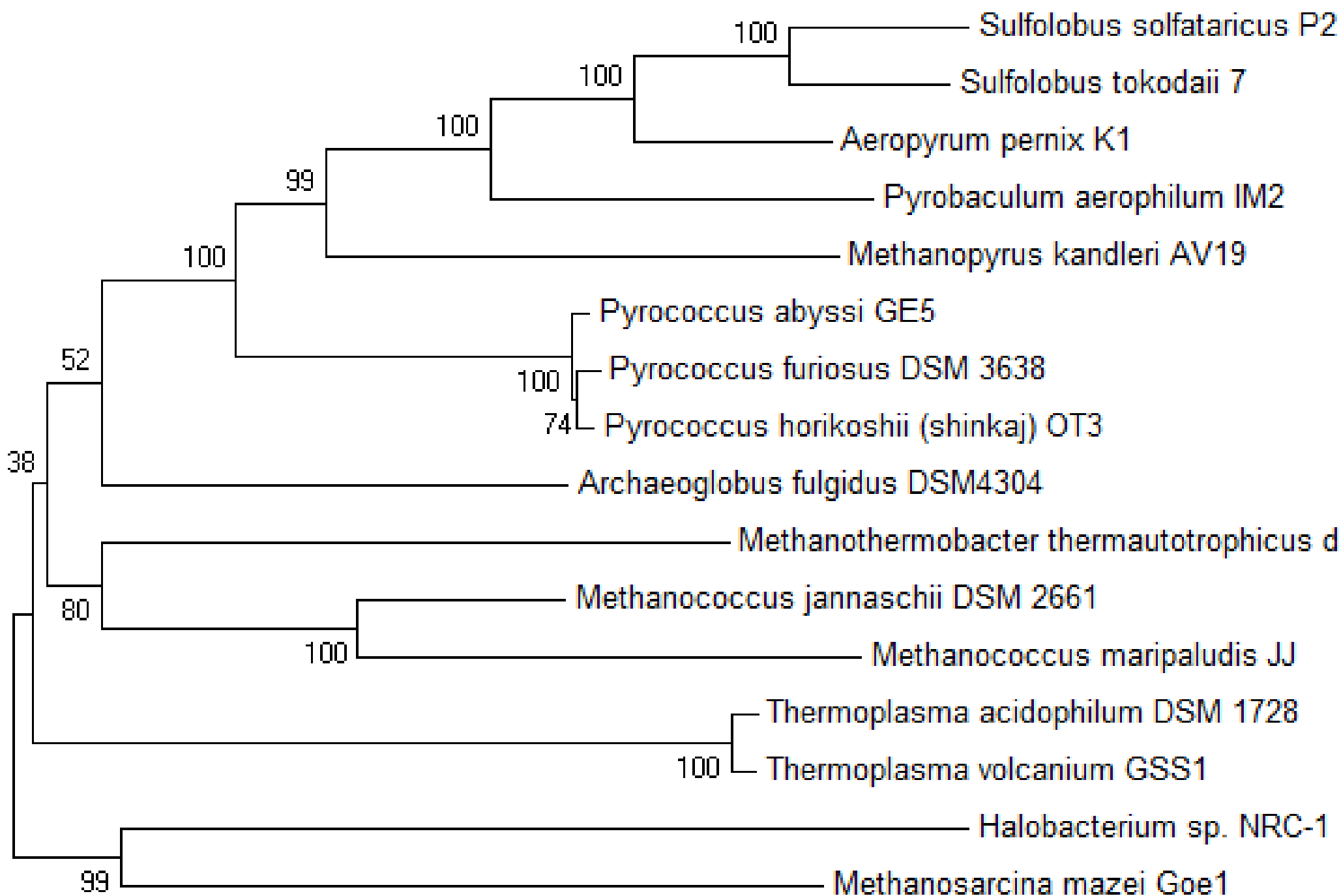

0.05

Supplement: Additional file 1 — Supplemental figure one. Archaea phylogenetic tree. Halobacterium sp. and Methanosarcina mazei were used as a composite internal outgroup to root the tree. Bootstrap % values are based on 10,000 replications. [file 1745-6150-4-4-S1.pdf]

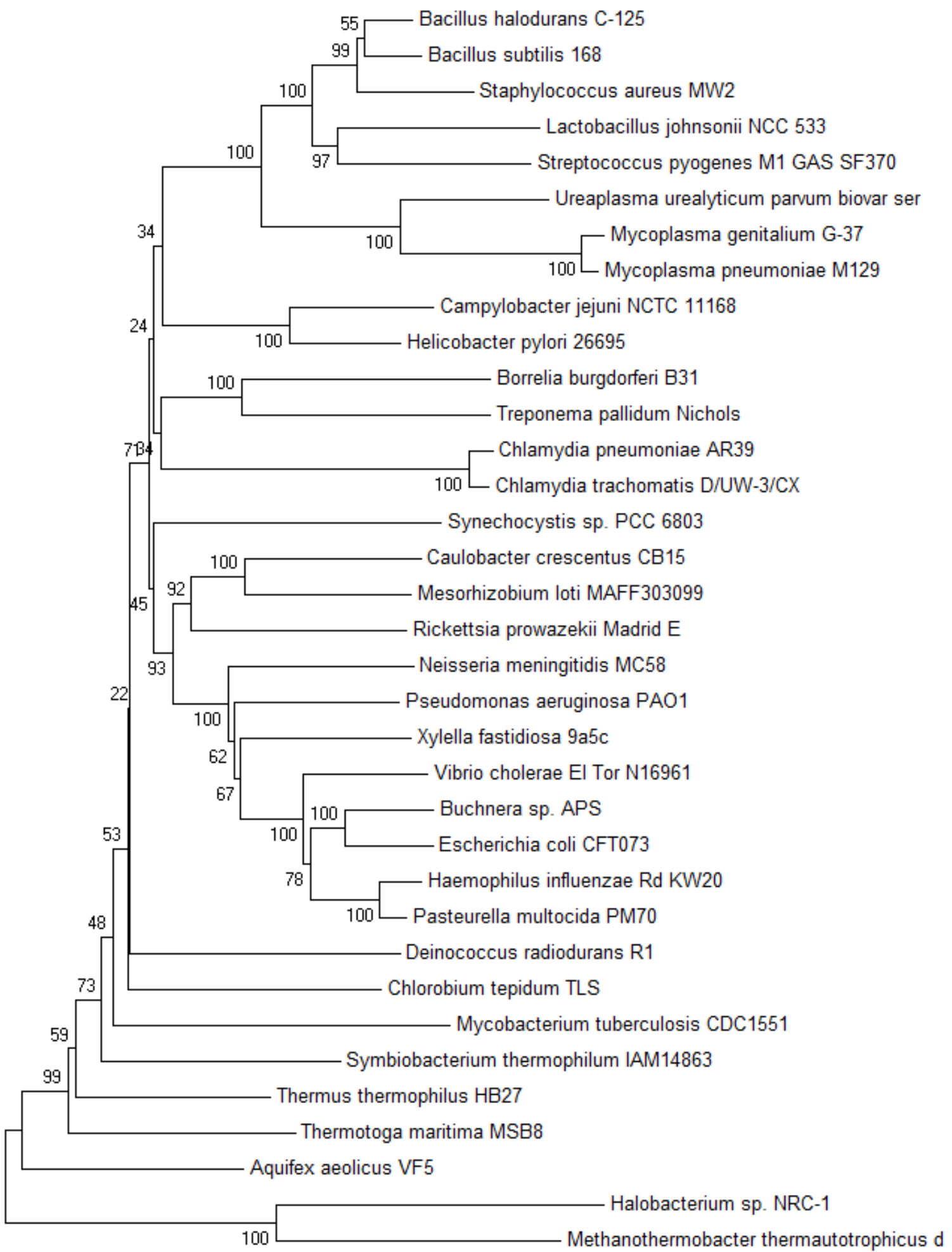

0.05

Supplement: Additional file 2 — Supplemental figure two. Bacteria phylogenetic tree. Halobacterium sp. and Methanothermobacter thermautotrophicus were used as a composite external outgroup to root the tree. Bootstrap % values are based on 10,000 replications. (The 35 species used to reconstruct the tree are the same used by Wolf et al in [21]. The full tree incorporating the sequences from 132 Bacteria species present in [18] is available from the authors upon request). [file 1745-6150-4-4-S2.pdf]

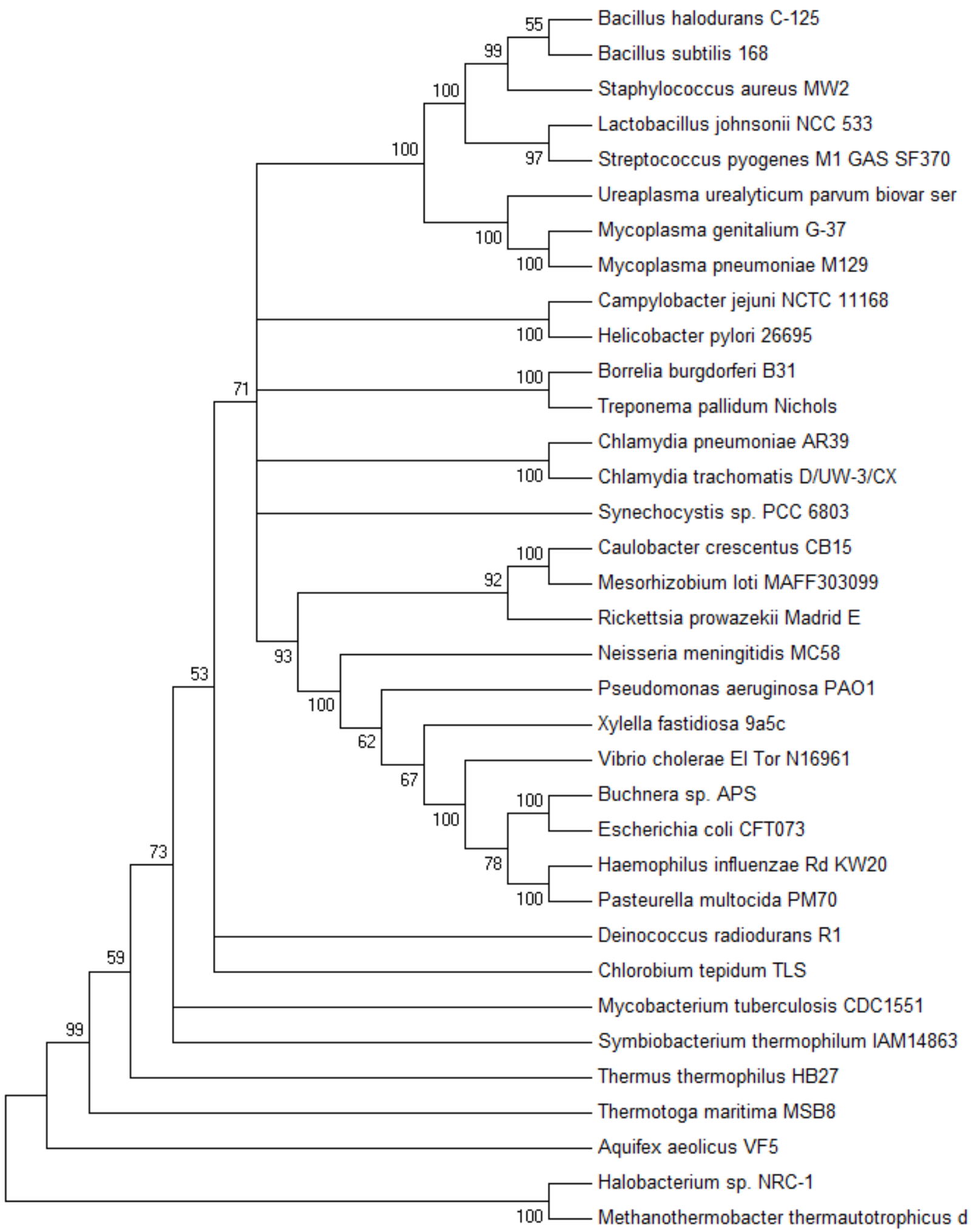

Supplement: Additional file 3 — Supplemental figure three. "Condensed" Bacteria phylogenetic tree. The internal tree branches not supported by bootstrap (i.e. with the bootstrap values less than 50%) were collapsed, resulting in multifurcations. This "condensed" tree topology was robust with respect to the phylogenetic reconstruction method and substitution model. [file 1745-6150-4-4-S3.pdf]

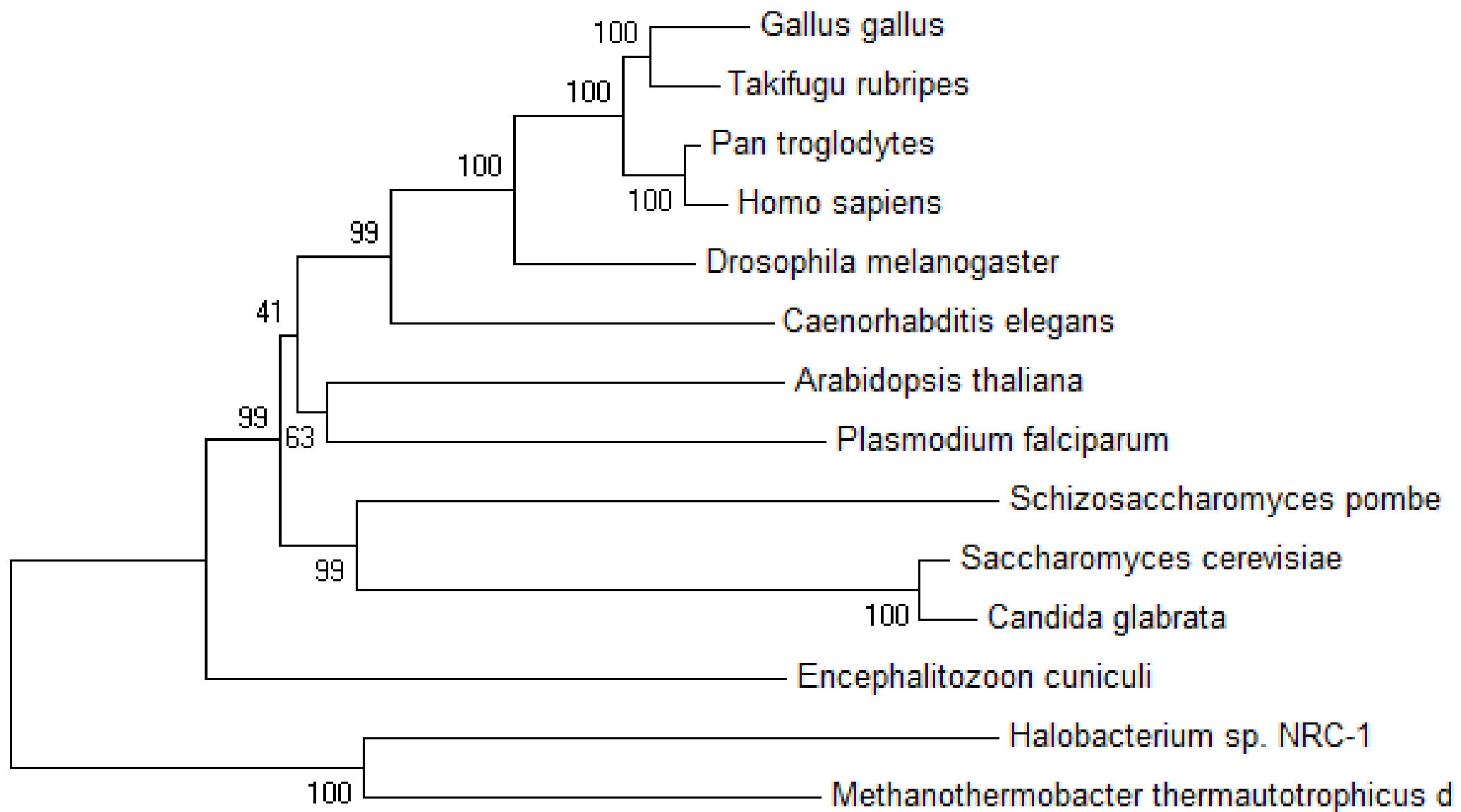

0.05

Supplement: Additional file 4 — Supplemental figure four. Eukarya phylogenetic tree. Halobacterium sp. and Methanothermobacter thermautotrophicus were used as a composite external outgroup to root the tree. Bootstrap % values are based on 10,000 replications. [file 1745-6150-4-4-S4.pdf]
