# Supplementary material for: One ancestor for two codes viewed from the perspective of two complementary modes of tRNA aminoacylation
Source: Biol Direct. 2009 Jan 27;4:4. doi: 10.1186/1745-6150-4-4 (PMC2669802; doi:10.1186/1745-6150-4-4)
Supplement: Additional file 5 — Supplemental figure five. One of the plausible models of elongation of the ancestral palindrome (see Fig. 5 in the text) to the tRNA cloverleaf. The model is based on duplications by self-priming and self-templating. The final cloverleaf has many of the sites of splitting tRNAs on minigenes in archaeal parasite Nanoarchaeum equitance 50 51, as well as the positions of processing in permuted tRNA genes from red algae Cyanidioschyzon merolae 52. [file 1745-6150-4-4-S5.doc]

Major units:

Anticodon triplets (5’-GCC-3’ and complementary 5’-GGC-3’)

Ending tetramers (5’-NCCA-3’and complementary 5’-UGGИ-3’)

**3’-ACCNCGGИGGU-5’**

Ancestral complementary palindromes

**5’-UGGИGCCNCCA-3’**

**3’-ACCNCGGИGGU…**

proto-acceptor

**||||||||**

Duplications

by self-priming and self-templating

**5’-*И*GCCNCCA…**

**3’-ACCNCGGИGGU…ACCNC**

proto-anticodon

**:||||||| || C**

**5’-*И*GCCNCCA…UGGИG**

**5’-uggиgccncca…ugg**

**3’-ACCNCGGИGGU…ACC…………NCCGИ…GGUACCNC**

**:||||||| ||| ||||| ||||| C**

**5’-*И*GCCNCCA…UGG…………ИGGCN…CCAUGGИG**

**3’-*n*cggиggu…acc**

**3’–ACCNCGGИGGU…ACC…gguaccnccgиgguNCCGИ-GGUACCNC**

**:||||||| ||| ||||||||||| ||||| ||||| C**

**5’-*И*GCCNCCA…UGG…ccauggиggcn ИGGCN-CCAUGGИG**

**C C N**

**A C**

**TψC**

**U C**

**G ? G**

**G - И**

**V**

**C - G**

**Acceptor**

**stem**

**C – G C C G**

**A – U N И C N**

**3’–A C C N C G G И G G U G G U A C C**

**Anticodon**

**arm**

**: | | | | | | | | | | | C**

***И* G C C N C C A U G N C C A U G G**

**G – C G И**

**C – G**

**C – G**

**A - И**

**U N**

**D**

**G C**

**G G**

**И G**
